# CALHM1/CALHM3 channel is intrinsically sorted to the basolateral membrane of epithelial cells including taste cells

**DOI:** 10.1038/s41598-019-39593-5

**Published:** 2019-02-25

**Authors:** Makiko Kashio, Gao Wei-qi, Yasuyoshi Ohsaki, Mizuho A. Kido, Akiyuki Taruno

**Affiliations:** 1 Department of Molecular Cell Physiology, Kyoto Prefectural University of Medicine, Kyoto, 602-8566 Japan; 2 JST, PRESTO, Kawaguchi, Saitama, 332-0012 Japan; 3 Department of Anatomy and Physiology, Saga University, Saga, 849-8501 Japan; 4 Present Address: Department of Physiology, Aichi Medical University, Nagakute, 480-1195 Japan

## Abstract

The CALHM1/CALHM3 channel in the basolateral membrane of polarized taste cells mediates neurotransmitter release. However, mechanisms regulating its localization remain unexplored. Here, we identified CALHM1/CALHM3 in the basolateral membrane of type II taste cells in discrete puncta localized close to afferent nerve fibers. As in taste cells, CALHM1/CALHM3 was present in the basolateral membrane of model epithelia, although it was distributed throughout the membrane and did not show accumulation in puncta. We identified canonical basolateral sorting signals in CALHM1 and CALHM3: tyrosine-based and dileucine motifs. However, basolateral sorting remained intact in mutated channels lacking those signals, suggesting that non-canonical signals reside elsewhere. Our study demonstrates intrinsic basolateral sorting of CALHM channels in polarized cells, and provides mechanistic insights.

## Introduction

Calcium homeostasis modulator 1 (CALHM1) and its homolog, CALHM3, hetero-hexamerize to form a non-selective fast-activating voltage-gated channel, CALHM1/CALHM3, which is permeable to large molecules including ATP^1^. CALHM1, which can form a slowly-activating voltage-gated channel by itself ^2–7^, has been reported to be expressed in various polarized cells including taste bud cells (TBCs)^8,9^, nasal^10^ and bladder^11^ epithelia, and cortical neurons^6,7,12^, and to mediate taste perception^8,13^ and memory formation^14^. Currently, CALHM1 is best characterized in TBCs. Taste buds face the oral cavity and underlying tissue, and detect taste compounds in foods and drinks. TBCs are polarized, with their apical and basolateral surfaces divided by tight junctions. Among distinct cell types, CALHM1 is expressed selectively in type II TBCs, which detect sweet, umami, or bitter compounds. In response to taste stimuli applied to the apical membrane, type II TBCs generate action potentials in the basolateral membrane, which lead to the release of ATP as the neurotransmitter towards gustatory nerves expressing the ATP-gated ion channel P2X2/3R^15^. The chemical synapse in type II TBCs, which lack conventional synaptic features including synaptic vesicles and expression of SNARE (soluble N-ethylmaleimide-sensitive factor attachment protein receptor) proteins, is unique in employing voltage-gated ion channels as conduits for neurotransmitter release. CALHM1 was identified as an essential, but not the sole, component of the neurotransmitter-release channel in type II TBCs^2^. Recently, a CALHM1/CALHM3 hetero-hexamer composed of CALHM1 and CALHM3 was identified as the ATP channel complex of type II TBC^1^. Another study reported CALHM1 localization in the basolateral membrane of type II TBCs at points of contact with P2X2R-expressing nerve fibers for the focal release of purinergic signals^16^. Although, mechanisms underlying CALHM1/CALHM3 localization in polarized cells such as taste cells remain unexplored.

Fully-matured membrane proteins that have undergone post-translational processing in the endoplasmic reticulum and Golgi are sorted to transporting vesicles and exported. In polarized epithelial cells, plasma membrane proteins are delivered into the apical or basolateral membrane, or both. For basolateral sorting, intrinsic basolateral transport signal sequences in intracellular domains are generally involved in recognition by adaptor proteins and subsequent sorting to clathrin-coated transporting vesicles^17^. Several canonical targeting sequences exist^18^. The most common types are tyrosine-based and dileucine motifs. Tyrosine-based motifs include YxxΦ (Y, tyrosine; x, any amino acid; and Φ, an amino acid with a bulky hydrophobic side chain)^19,20^ and NPxY (N, asparagine; and P, proline)^21^. Dileucine motifs consist of diverse hydrophobic amino acids (LL, IL, LEL, and ML)^19,22^. Rarer motifs include those with a single leucine^23–25^ and one with a polyproline core^22^.

Herein, we generated an antibody against a short peptide sequence corresponding to the carboxyl terminal end of mouse CALHM1, and data from immunohistological analyses using it supported punctate localization near nerve fibers in the basolateral membrane of type II TBCs^16^. As plasma membrane proteins cannot diffuse over the tight junction, CALHM1/CALHM3 must initially be delivered to the basolateral membrane, and subsequently accumulate at points of contact with nerve fibers. Here, using an epithelial model of MDCKII cells, we explored the mechanisms of the polarized sorting of CALHM1/CALHM3 to further understanding of the structural basis behind the regulation of CALHM channel localization in polarized cells.

## Results

### CALHM1 localization in taste bud cells

**Figure 1:**
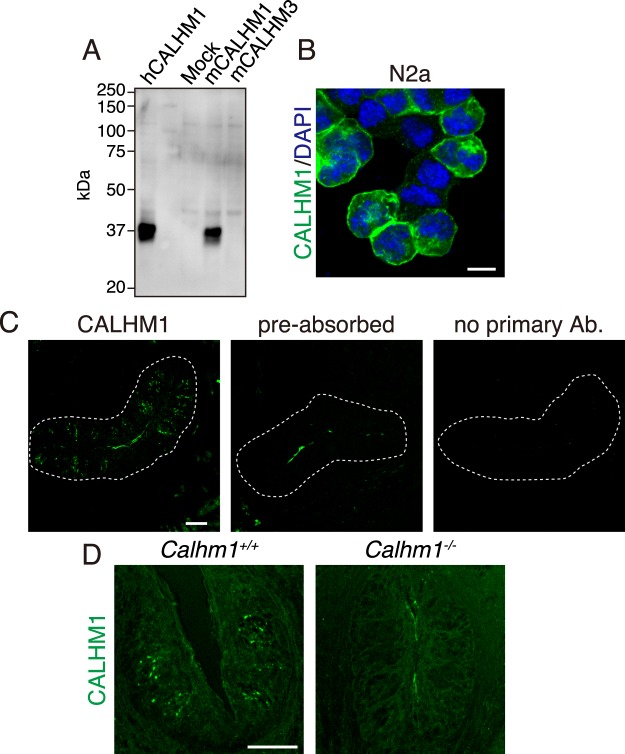
The specificity of the CALHM1 antibody. (**A**) The specificity of the obtained CALHM1 antibody was verified by Western blotting using 40 µg of whole-cell lysates obtained from N2a cells transfected with empty vector (Mock), mouse CALHM1 (mCALHM1), mouse CALHM3 (mCALHM3), or human CALHM1 (hCALHM1). (**B**) Immunofluorescence confocal images of N2a cells transiently transfected with CALHM1 and 3. CALHM1 and DAPI are labeled green and blue, respectively. Scale bar, 10 µm. (**C**) Confocal images (green) of the circumvallate papilla immunostained with the CALHM1 antibody, immunizing peptide-preabsorbed CALHM1 antibody, or no primary antibody. (**D**) Immunofluorescence confocal images (green) of the circumvallate papilla of wild-type (*Calhm1*^+/+^) and CALHM1 knockout (*Calhm1*^−/−^) mice with the CALHM1 antibody. Punctate intragemmal signals were observed in *Calhm1*^+/+^ but not *Calhm1*^−/−^ mice, supporting the specificity of the antibody and revealing punctate CALHM1 localization in the taste buds. Scale bar, 50 µm. Data are representative of circumvallate papillae in more than three animals.

**Figure 2:**
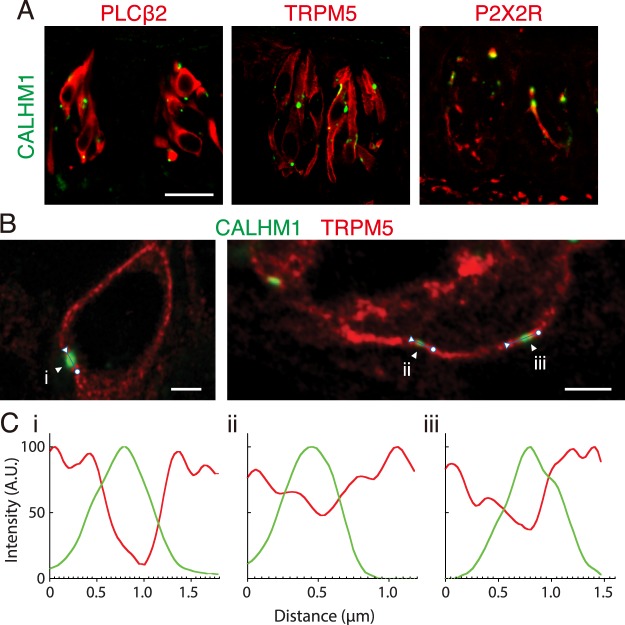
Localization of CALHM1 in the basolateral membrane of type II TBCs close to afferent nerve fibers. (**A**) Confocal images of double-staining of CALHM1 (green) and PLCβ2, TRPM5, or P2X2R (red) in the circumvallate taste buds. Scale bar, 20 µm. Data are representative of multiple taste buds from each circumvallate papilla in three animals. (**B**) Airyscan images of TBCs in the circumvallate taste buds double-stained for CALHM1 (green) and TRPM5 (red). Scale bar, 2 µm. Data are representative of five images. (**C**) Line plots of basolateral membrane areas carrying a single CALHM1 punctum shown in (**C**) (i–iii). CALHM1 signal, green line; TRPM5 signal, red line; A.U., arbitrary units.

Immunofluorescence staining of tongue sections containing circumvallate papillae using an antibody targeting the Cter end of mouse CALHM1 (Fig. 1A,B) revealed small punctate signals within the wild-type taste buds (Fig. 1C,D). The immunoreactivities are specific to CALHM1 because they were absent in *Calhm1* knockout mice (Fig. 1D), with the immunizing peptide-preabsorbed antibody, and in the absence of the primary antibody (Fig. 1C). Almost all CALHM1 signal puncta were associated with type II TBC marker proteins PLCβ2 and TRPM5, confirming CALHM1’s selective expression in type II TBCs (Fig. 2A). To examine the relationship between CALHM1 and the basolateral membrane, we performed high-resolution imaging of TBCs immunostained with antibodies against CALHM1 and TRPM5. TRPM5 is distributed throughout the basolateral membrane but not in apical microvilli^26^. CALHM1 signals were observed in the basolateral membrane lined by TRPM5 immunoreactivity (Fig. 2B). Similar results were obtained in TBCs double-stained with antibodies against CALHM1 and KCNQ1, a basolateral membrane marker for all TBCs (Supplementary Fig. S1). CALHM1 signals were absent on the apical surface of TBCs. Together with the fact that CALHM1/3 channel currents have been recorded by whole-cell voltage-clamp recordings^1,2^, these observations indicate that CALHM1 is present in the basolateral membrane. Furthermore, each CALHM1 punctum was localized close to P2X2R-expressing afferent gustatory nerve fibers (Fig. 2A), as recently reported by Romanov *et al*.^16^. These results strongly support the punctate CALHM1 localization in the basolateral membrane of type II TBCs at points of contact with nerve fibers. Notably, high-resolution imaging revealed that immunoreactivities for TRPM5 and KCNQ1 reduced or disappeared in plasma membrane patches with CALHM1 immunoreactivity (Fig. 2C and Supplementary Fig. S1).

### Basolateral sorting of CALHM1/CALHM3 in epithelial cells

**Figure 3:**
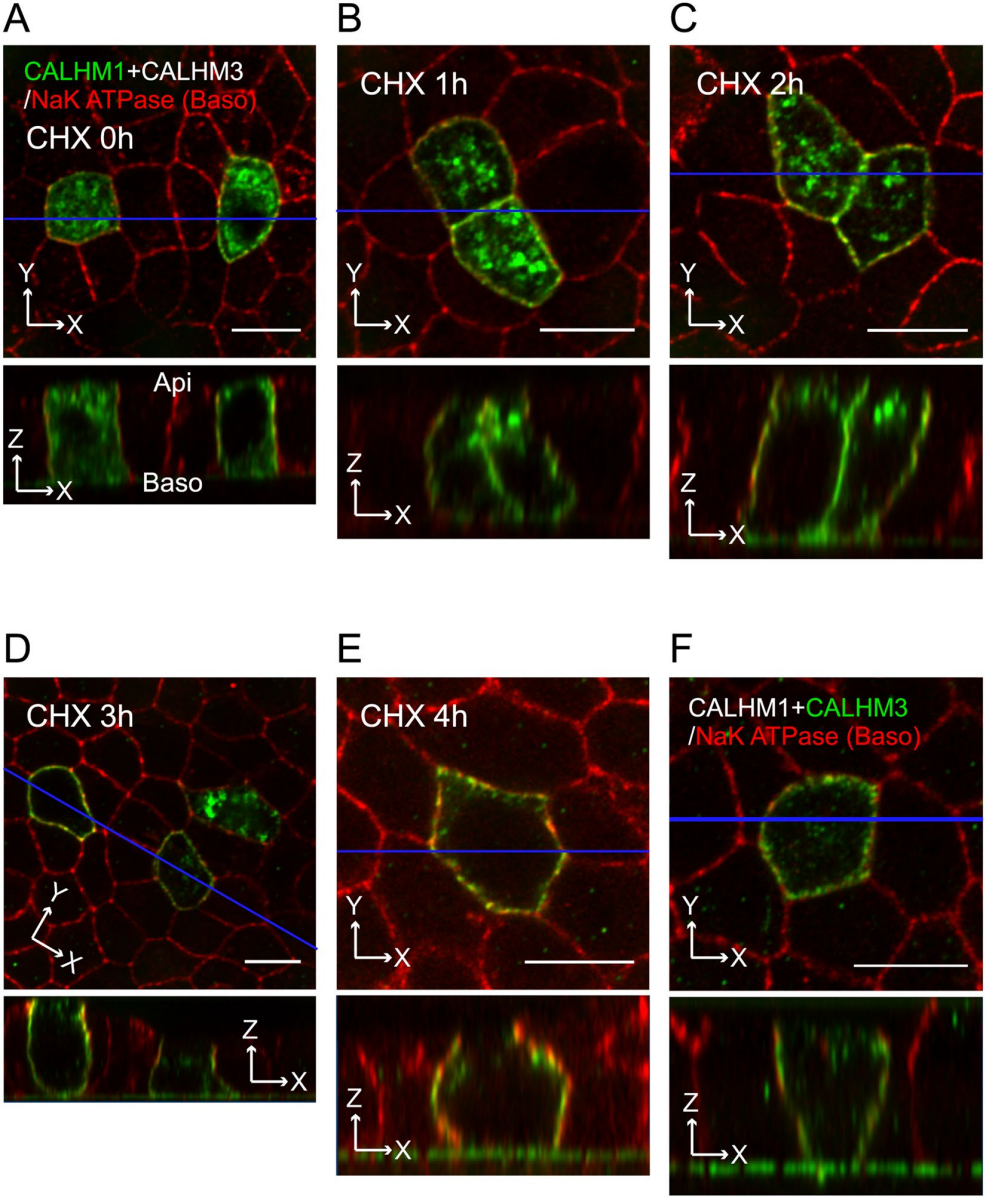
Localization of CALHM1/CALHM3 in the basolateral membrane of MDCKII epithelia. (**A–E**) Fluorescence double-labeling of GFP (green) and Na^+^/K^+^ ATPase (red) in polarized MDCKII cells that were transiently transfected with CALHM1-GFP and untagged CALHM3 and treated with 100 µg/mL cycloheximide (CHX) for the indicated time. (**F**) Double-staining of GFP (green) and Na^+^/K^+^ ATPase (red) in MDCKII epithelia transfected with untagged CALHM1 and CALHM3-GFP and treated with CHX for 2 h. Na^+^/K^+^ ATPase signals were used as a marker for the basolateral membranes. Upper panel, XY image. Lower panel, XZ image reconstructed at the blue line in the upper image. Note that Transwell filter membranes exhibit autofluorescence in the green channel and generate straight signals at the bottom of the cells in the XZ images throughout the manuscript. Api, apical membrane; Baso, basolateral membrane; Scale bars, 10 µm. Data are representative of two experiments.

We examined the direction of CALHM1/CALHM3 sorting in the apical-basolateral axis in polarized MDCKII cells, a well-established platform for the analysis of epithelial protein sorting^27^, in which neither CALHM1 nor CALHM3 are endogenously expressed. Transient transfection of CALHM1-GFP and untagged CALHM3 was performed because the generation of cells stably expressing CALHM1 was unsuccessful due to the cytotoxicity of CALHM channel expression^2,3^. We subsequently immunostained CALHM1-GFP and Na^+^/K^+^ ATPase, a basolateral membrane marker protein, and acquired z-stack images of the stained cells. CALHM1 was primarily observed within the cytoplasm with a granular appearance, while a small fraction was noted in the Na^+^/K^+^ ATPase-positive basolateral membrane (Fig. 3A). CHX treatment decreased the number of intracellular granular signals over time, and signals only remained in the basolateral membrane at 4 h, with none in the putative apical membrane region between the apical ends of Na^+^/K^+^ ATPase signals (Fig. 3B–E). As expected from the heteromer formation between CALHM1 and 3^1^, when untagged CALHM1 and CALHM3-GFP were expressed, CALHM3 signals were also observed only in the basolateral membrane (Fig. 3F). These observations suggest that the cytoplasmic granular signals were transporting vesicles containing newly synthesized CALHM1/CALHM3 complexes that were being trafficked to the basolateral membrane. Therefore, only in subsequent immunostaining experiments, we used 2-h CHX-treatment to facilitate the detection of CALHM1 in the plasma membrane. Any reduction in CALHM1 protein levels caused by longer treatment would impair analysis. When CALHM1 was expressed in the absence of CALHM3, its plasma membrane localization was indistinguishable from cytoplasmic signals even after prolonged CHX treatment (data not shown), possibly due to the slow forward trafficking^1^.

**Figure 4:**
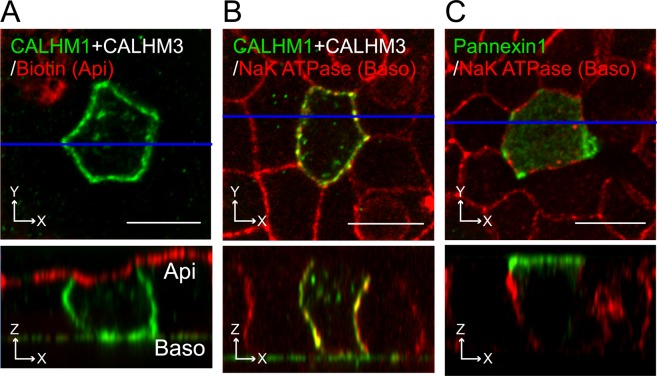
Distinct membrane localization of CALHM and pannexin channels. (**A–B)** Fluorescence double-labeling of polarized MDCKII cells transfected with CALHM1-GFP and untagged CALHM3. GFP (green) and apical membrane-bound biotin (**A**) or Na^+^/K^+^ ATPase (**B**) (red) were double-stained. Biotin and Na^+^/K^+^ ATPase signals were respectively used as markers for the apical and basolateral membranes. (**C**) Double-staining of GFP (green) and Na^+^/K^+^ ATPase (red) in MDCKII epithelia transfected with pannexin 1-GFP. Upper panel, XY image. Lower panel, XZ image reconstructed at the blue line in its upper image. Api, apical membrane; Baso, basolateral membrane; Scale bars, 10 µm. Data are representative of four experiments.

Furthermore, the apical membrane was labeled by biotinylation. Figure 4A,B clearly demonstrate that CALHM1 signals are co-localized with Na^+^/K^+^ ATPase but not with apical biotin signals, confirming the absence of CALHM1/CALHM3 in the apical membrane. This suggests that either CALHM1 or 3, or both, possess signal sequences responsible for basolateral sorting of the CALHM1/CALHM3 complex. In contrast, the pannexin 1 channel, sharing functional and structural similarities with CALHM^4,8^ but functions in the apical membrane of various epithelia^28,29^, was selectively localized in the apical membrane of MDCKII epithelia, suggesting distinct sorting mechanisms between these channels (Fig. 4C).

### Basolateral sorting signals in CALHM1 and CALHM3

**Figure 5:**
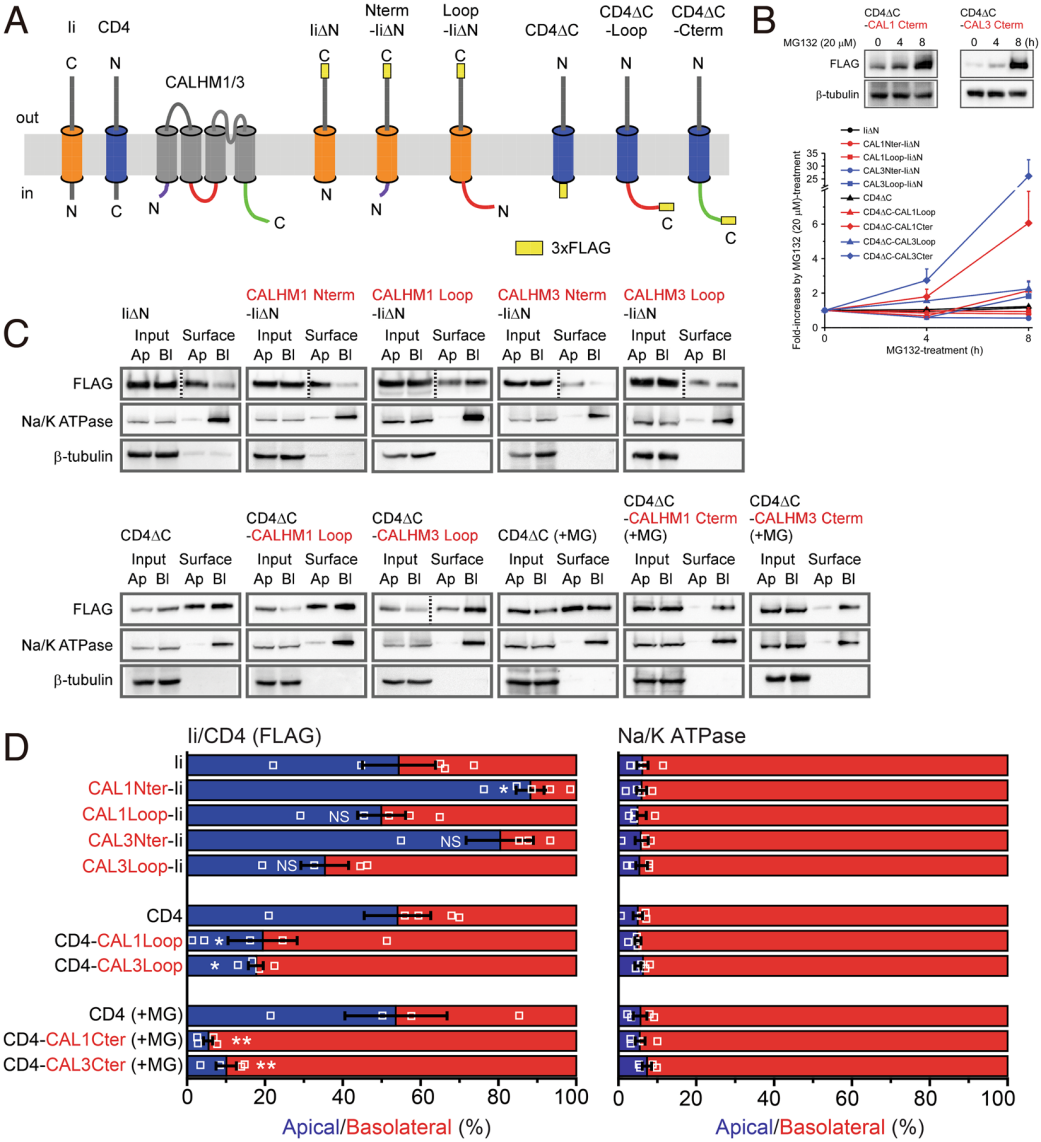
Loop and Cter of CALHM1 and 3 contain basolateral sorting signals. (**A**) Illustration of chimeric constructs of the intracellular domain-truncated invariant chain (IiΔN) or CD4ΔC and an intracellular domain of mouse CALHM1 or 3. All chimeras were FLAG-tagged on their Cter. (**B**) Effects of MG132 (20 µg/mL) on expression levels of the chimeric proteins. Upper panels display representative Western blots of CD4ΔC-CALHM1Cter and CD4ΔC-CALHM3Cter, and the lower panel shows a quantitative summary of expression levels of each chimera 0, 4, and 8 h after MG132 treatment. N = 3. Uncropped blots are shown in Supplementary Fig. S2. (**C**) Representative Western blots of the domain-specific biotinylation assays in stable MDCKII clones expressing each chimera. Whole-cell lysates (Input) of apically (Ap)- or basolaterally (Bl)-biotinylated cells and their avidin pull-down eluates (Surface) were analyzed by SDS-PAGE/Western blotting. FLAG-tagged chimeric proteins were detected by anti-FLAG antibody. Na^+^/K^+^ ATPase and β-tubulin were detected as markers of the basolateral membrane and cytosol, respectively. IiΔN and CD4ΔC alone were tested as controls. CD4ΔC-CALHM1Cter and CD4ΔC-CALHM3Cter were analyzed after 4-h MG132 treatment (+MG). Images taken from the same gel but at different exposures are separated by dashed black lines. Uncropped blots are shown in Supplementary Fig. S3. (**D**) The amounts of each chimera detected in the apical and basolateral membranes are expressed as percentages of the total amount in the entire plasma membrane. N = 4~5. ^*NS*^*p* > 0.05, *p < 0.05 and **p < 0.01 (vs. control, Bonferroni post-hoc test).

Both CALHM1 and 3 are four-transmembrane proteins with cytoplasmic Nter, Cter and Loop. To identify intracellular domains containing basolateral sorting signals, we utilized two single membrane-spanning proteins, CD4 and Ii, which have opposite membrane topologies with their Cter and Nter in the cytoplasm, respectively (Fig. 5A). We created a series of FLAG-tagged chimeric constructs of a recipient protein (Ii or CD4) and an intracellular region of CALHM1 or 3 (Fig. 5A), and established MDCKII clones stably expressing one of those chimeras. Ii Nter was replaced with Nter or Loop of CALHM1 or 3, and CD4 Cter with Cter or Loop of CALHM1 or 3, while IiΔN and CD4ΔC were used as controls. Loop was fused with both IiΔN and CD4ΔC because the direction and/or distance from the plasma membrane of certain sorting signal sequences is critical^25^. Apical and basolateral sorting of the eight chimeras were quantified in polarized epithelia of the stable clones by the domain-specific surface biotinylation assay. Because protein levels of CD4ΔC-CALHM1 Cter and CD4ΔC-CALHM3 Cter were too low for quantitative analysis but were rescued by treatment with MG132, a proteasome inhibitor (Fig. 5B), only Cter chimeras were analyzed after 4-h treatment with MG132. Representative Western blots and quantitative results of the domain-specific surface biotinylation assay are shown in Fig. 5C,D. The amounts of each chimera detected in the apical and basolateral membranes are expressed as percentages of the total amount in the entire plasma membrane. The control proteins lacking intracellular domains, IiΔN and CD4ΔC, did not show selective sorting, as demonstrated by equal distribution between the apical and basolateral membranes. Regarding chimeras, none of Nter and Loop of CALHM1 and 3 promoted basolateral delivery of IiΔN, and CALHM1 Nter increased apical delivery of IiΔN. In contrast, Cter and Loop of CALHM1 and 3 markedly promoted basolateral sorting of CD4ΔC. These results demonstrate that Cter and Loop of CALHM1 and 3 contain intrinsic basolateral sorting signals.

### Conserved canonical basolateral sorting signals in CALHM1

**Figure 6:**
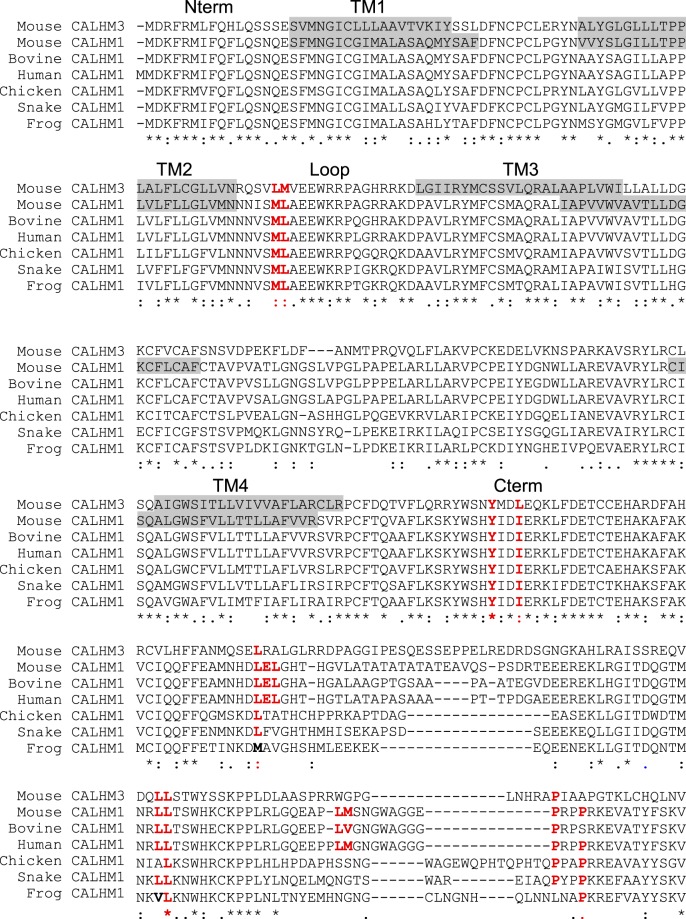
Alignment of primary sequences of mouse CALHM3 and CALHM1 from different vertebrates. Putative transmembrane domains of mouse CALHM1 and 3 are indicated by gray shades and conserved canonical basolateral sorting motifs are highlighted by red letters. *Represents a fully conserved residue, and ‘:’ and ‘.’ indicate strong and weak similarities, respectively.

We identified conserved canonical basolateral sorting motifs in the intracellular domains of CALHM1 and 3 (Fig. 6): CALHM1 motifs: one tyrosine-based, four dileucine, and one proline-based in Loop and Cter but none in Nter; CALHM3 motifs: dileucine in Loop and tyrosine-based and one dileucine (LL) in Cter.

**Figure 7:**
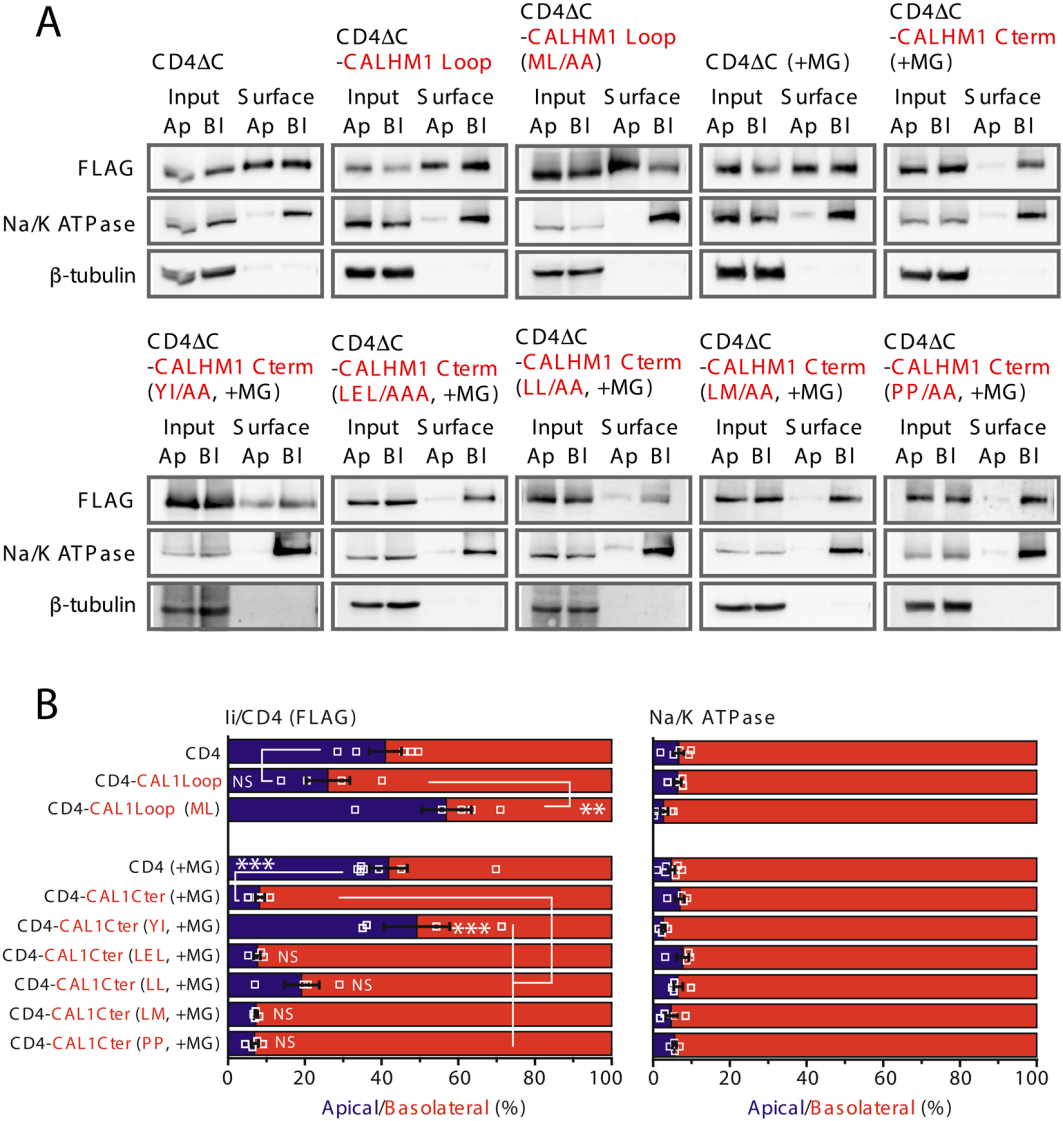
Identification of functional basolateral sorting signals. (**A**) Representative Western blots of the domain-specific biotinylation assays in stable clones of MDCKII cells expressing CD4ΔC, CD4ΔC-CALHM1Loop, CD4ΔC-CALHM1Cter, or either one of the mutant chimeras in which one of the candidate basolateral sorting signals was disrupted by alanine substitution. Whole-cell lysates (Input) of apically (Ap)- or basolaterally (Bl)-biotinylated cells and their avidin pull-down eluates (Surface) were analyzed by Western blotting. CD4ΔC fusion proteins were detected by anti-FLAG antibody. Na^+^/K^+^ ATPase and β-tubulin signals serve as markers for the basolateral membrane and cytosol, respectively. CD4ΔC-CALHM1Cter proteins were analyzed after 4-h MG132 treatment (+MG). Uncropped blots are shown in Supplementary Fig. S4. (**B**) The amounts of each chimera detected in the apical and basolateral membranes are expressed as percentages of the total amount in the entire plasma membrane. N = 4~7. ^*NS*^*p* > 0.05 **p < 0.01 and ***p < 0.001 (Bonferroni post-hoc test).

To identify functional signals, alanine mutations were introduced to those canonical motifs in CALHM1 and chimeric constructs of CD4ΔC, creating mutant Loop or Cter. After establishing MDCKII clones stably expressing the mutant chimeras, we quantified their polarized sorting by the domain-specific surface biotinylation assay (Fig. 7A,B). Disruption of the dileucine motif (ML/AA) in Loop and tyrosine-based motif (YI/AA) in Cter significantly decreased basolateral sorting of CD4ΔC-Loop and CD4ΔC-Cter, respectively. Mutation of a dileucine motif in Cter (LL/AA) also slightly reduced basolateral sorting of CD4ΔC-Cterm (non-significant). In contrast, disruption of other motifs in Cter (LEL, LM, and PP) had no effect. These results identify the dileucine motif in Loop (ML), the tyrosine-based motif in Cter (YIDI), and potentially one dileucine motif in Cter (LL) as functional signals for basolateral sorting of CALHM1. CALHM3 also conserves the three motifs in Loop and Cter, and no other unique canonical motifs exist (Fig. 6), suggesting that these conserved motifs in CALHM3 are also functional.

### Canonical signal motifs are not necessary for basolateral sorting of CALHM1/CALHM3

**Figure 8:**
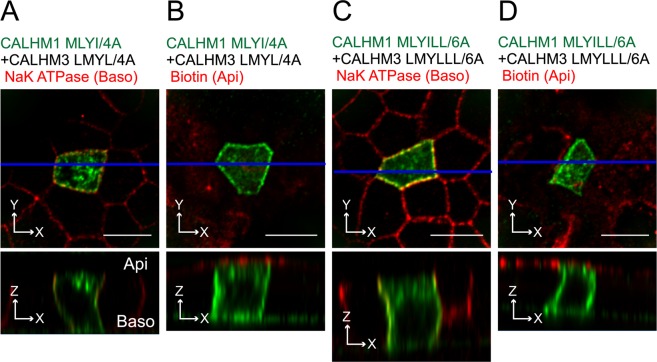
Canonical signals are non-essential for basolateral sorting of CALHM1/CALHM3 channel. (**A–B)** Fluorescence double-labeling of polarized MDCKII cells expressing GFP-fused CALHM1 MLYI/4A and untagged CALHM3 LMYL/4A with GFP (green) and Na^+^/K^+^ ATPase (**A**) or apical membrane-bound biotin (**B**) (red). (**C–D**) MDCKII epithelia expressing GFP-fused CALHM1 MLYILL/6A and untagged CALHM3 LMYLLL/6A were double-stained with GFP (green) and Na^+^/K^+^ ATPase (**C**) or apical membrane-bound biotin (**D**) (red). Upper panel, XY image. Lower panel, XZ image reconstructed at the blue line in the upper image. Api, apical membrane; Baso, basolateral membrane; Scale bars, 10 µm. Data are representative of three experiments.

Finally, the Loop dileucine and Cter tyrosine-based motifs in CALHM1 and 3 were substituted with alanines, the resulting mutants CALHM1 (MLYI/4A) and CALHM3 (LMYL/4A) were used to transiently co-transfect polarized MDCKII cells, and CALHM1’s distribution was evaluated by immunostaining. The domain-specific surface biotinylation assay was infeasible because generation of stable cell lines expressing full-length CALHM1 proteins was unsuccessful due to their cytotoxicity^2,3^. Disruption of these motifs did not alter basolateral sorting of the channel (Fig. 8A,B). Additional mutations of the conserved dileucine motifs (LL) in Cter were introduced into CALHM1 and 3, and the resulting mutant CALHM1/3 channel composed of CALHM1 (MLYILL/6A) and CALHM3 (LMYLLL/6A) still maintained basolateral sorting (Fig. 8C,D), suggesting that the canonical basolateral sorting signals identified by the chimeric approach are dispensable for basolateral delivery of the actual CALHM1/CALHM3 channel complex.

## Discussion

Recently, in type II TBCs, CALHM1 (*i.e*., CALHM1/CALHM3 channel^1^) was located in the basolateral membrane in discrete puncta, localized close to P2X2/3 receptor-expressing afferent nerve fibers^16^. Furthermore, large mitochondria are consistently juxtaposed closely to membrane areas accumulating CALHM1 to provide ATP^16^. The close apposition of the ATP source, ATP-release channel, and ATP receptors is thought to enable regulated, spatially-localized ATP release without synapses. That study^16^ employed monoclonal antibody mapping to residues 318 to 328 (EEPPLMGNGWA) on Cter of human CALHM1, corresponding to 321–330 (EAPLMSNGWA) of mouse CALHM1. Our independent immunohistological data using a polyclonal antibody against the Cter end (residues 338–348) of mouse CALHM1 generated in this study support the punctate localization of CALHM1 in the basolateral membrane of type II TBCs specialized for focal neurotransmitter release. Our data also suggest that membrane patches carrying CALHM1, which are sites for neurotransmission, are distinct in terms of the local protein repertoire from the remaining basolateral membrane, which expresses TRPM5 and KCNQ1 and contributes to general cellular signaling. This is a novel insight into functional membrane micro-domains within type II TBCs.

How is the localization of CALHM1/CALHM3 regulated? We hypothesized two processes: As it cannot cross the tight junction, it needs to be delivered to the basolateral membrane first, and then accumulates at points of contact with nerve fibers. This work addressed the initial polarized sorting of the channel in an epithelial model. In polarized MDCKII cells, CALHM1/CALHM3 was distributed throughout the basolateral membrane, suggesting intrinsic basolateral sorting of the channel in epithelial cells. TBCs must contain additional machinery to further accumulate CALHM1/CALHM3 in localized spots.

In chimeric experiments, Loop and Cter of both subunits induced basolateral sorting of the recipient membrane proteins. We identified a dileucine motif in Loop and a tyrosine-based motif in Cter that are responsible among conserved basolateral sorting motifs, while a conserved dileucine motif in Cter may also play a role. Of note, the dileucine motif in Loop was functional only when Loop was fused with CD4ΔC but not IiΔN, suggesting that the direction and/or distance of the motif from the membrane surface is critical for functionality. Furthermore, CALHM1 is degraded by the ubiquitin-proteasome system^3^. The MG132 sensitivity of CD4ΔC-CALHM1 Cter and CD4ΔC-CALHM3 Cter but not the other chimeras suggests that ubiquitination sites reside in Cter of these subunits.

Sorting mechanisms in multipass membrane proteins are less clear and more complex compared with those in single membrane-spanning proteins. CALHM1/CALHM3 is a hetero-hexamer with unknown subunit stoichiometry, and each subunit has four transmembrane domains. Surprisingly, the disruption of all intracellular signal motifs in CALHM1 and 3 found in the chimeric protein experiments failed to alter the basolateral delivery of the CALHM1/3 channel complex, suggesting that those canonical signals are partly responsible but additional signals are also involved. As reported in Kir2.1, which forms a homo-tetrameric channel with each subunit exhibiting two transmembrane domains^30^, the tertiary and quaternary structures of CALHM1/CALHM3 may form a “signal patch” within its intra- or inter-subunit assembly. Intracellular Nter and Cter of Kir2.1 together form a Golgi export signal interacting with AP1, which is also engaged in basolateral sorting^18^. The structure and subunit stoichiometry of the CALHM1/CALHM3 complex would thus provide further insight into its sorting mechanisms. Alternatively, the CALHM1/3 channel may bind with an unknown protein that transports it to the basolateral membrane.

CALHM1 has been reported to be expressed in brain neurons and involved in memory formation^14^ and the pathogenesis of Alzheimer’s disease^7^. However, it remains unclear where CALHM1 is localized in neurons and whether CALHM3 is involved. Like epithelial cells, cortical neurons have a highly polarized structure with an axon at one end and dendrites at the other. Epithelial cells and neurons utilize common machineries to sort proteins^31^. Basolateral membrane proteins in epithelial cells are localized in the cell body and dendrites in neurons, whereas apical proteins are in axons. Therefore, CALHM channels may be localized in the cell body and/or dendrites in neurons. A recent study^11^ reported that CALHM1 is expressed in the urothelium as well as suburothelium and detrusor muscle of the porcine bladder, and that it mediates hypotonic stretch-induced ATP release from those cells in culture. Both the apical and basolateral membranes of urothelial cells exhibited marked immunoreactivity in response to a commercially available CALHM1 antibody (HPA018317, Sigma), whose specificity for CALHM1 has not been validated using *Calhm1* knockout tissues. However, our specificity-validated CALHM1 antibody did not generate signals in mouse urothelial cells (data not shown). This may reflect species differences or a lack of specificity for CALHM1 of the commercial antibody. Another recent study^10^ reported that CALHM1 is involved in mechanical stress-induced ATP release from the apical membrane of cultured mouse nasal epithelium. Although that study provides compelling functional evidence using *Calhm1* knockout mice, CALHM1 localization was not tested. Reliable histological analyses are indispensable.

Overall, the present results, revealing intrinsic basolateral targeting of the CALHM1/CALHM3 channel, provide a basis for future research on molecular mechanisms leading to spatial confinement of the CALHM channel function not only in TBCs but also other polarized cells, including neurons.

## Materials and Methods

### Ethics

All experiments were conducted using protocols approved by the Institutional Animal Care and Use Committee of Kyoto Prefectural University of Medicine.

### CALHM1 antibody

Guinea pig polyclonal anti-mouse CALHM1 antibody was raised against a peptide corresponding to the carboxyl terminal end of mouse CALHM1 (residues 338 to 348: RKEVATYFSKV) and affinity-purified with the peptide. N2a cells transfected with CALHMs^3^ were used to assess antibody specificity. In Western blots, this antibody reacted with mouse and human CALHM1 but not mouse CALHM3 (Fig. 1A), and it generated immunofluorescence signals in CALHM1-transfected (Fig. 1B) but not mock-transfected (data not shown) cells, supporting its specificity for CALHM1.

### Immunohistochemistry

Nine micrometer-thick fixed sections of tongue epithelia containing circumvallate papillae were prepared from wild-type or *Calhm1* knockout mice, as previously described^2,3^. Sections were treated in a preheated target retrieval solution (Agilent Technologies) at 80 °C for 20 min and blocked in PBS containing 5% normal donkey serum and 0.1% Triton X-100. As primary antibodies, guinea pig anti-CALHM1 (1:10,000), rabbit anti-TRPM5 (Alomone), rabbit anti-PLCβ2 (Santa Cruz), rabbit anti-P2X2R (Sigma), goat anti-KCNQ1 (Santa Cruz) antibodies were used. For fluorescent labeling of CALHM1, signals were developed using biotin-conjugated donkey anti-guinea pig IgG antibody (Millipore) followed by an avidin-biotin complex (Vector Laboratories), a tyramide signal amplification biotin system (Tyramide SuperBoost Kit, Thermo Fisher), and Alexa fluor 488-conjugated streptavidin (Thermo Fisher). Other proteins were simultaneously labeled using Alexa fluor 546-conjugated donkey anti-rabbit IgG or Alexa fluor 647-conjugated donkey anti-goat IgG (Thermo Fisher). Images were acquired using a LSM510 confocal scanning microscope (Carl Zeiss) with an EC Plan-Neofluar 20×/0.50 NA objective or an EC Plan-Neofluar 40×/1.30 NA oil objective with the pinhole set to 2 Airy Units for the green channel and adjusted to yield the same optical slice thickness in the red channel. Images show single optical sections. DAPI was excited with 2 photons using the MaiTai titanium-sapphire laser tuned at 780 nm (Spectra-Physics). Some of the images shown in Fig. 2B and Supplementary Fig. S1 were acquired using a Zeiss Axio Observer Z1 with Plan Apochoromat 63×/1.40 NA oil DIC M27 and an LSM 800 confocal unit with Airyscan module (Carl Zeiss). The fluorescence was excited using laser lines of 640, 561, and 488 nm and each detection wavelength was 643–700, 563–640, and 491–575 nm through main beam splitters of 640 (T10/R90), 561, and 488, respectively. The images were Airyscan processed automatically with an additional manual adjustment of plus 0.5 per channel using the Zeiss Zen Blue 2.3 software package.

### Plasmid vectors

Mouse CALHM1 (Accession# NM_001081271) and CALHM3 (Accession# XM_140729) cDNAs were gifts from Dr. J. Kevin Foskett (University of Pennsylvania). Human CD4 (Accession# M12807.1)^32^ and human invariant chain (Ii, Accession# NM_004355.3) with the tetramerization motif mutated (Q63A/T56A/T66A)^33^ were provided by Dr. Gerald W. Zamponi (University of Calgary) and Dr. Gergely Lukacs (McGill University), respectively. The membrane topology of CALHM1 with four transmembrane domains and intracellular carboxyl terminus (Cter) and amino terminus (Nter) was experimentally determined^4^. A similar topology of CALHM3 was predicted using TMHMM v.2.0. Nter, intracellular loop (Loop), and Cter of CALHM1 and 3 are: in CALHM1, M1-E16 (Nter), N72-A107 (Loop), S203-V349 (Cter); in CALHM3, M1-E16 (Nter), R72-D91 (Loop), P203-V348 (Cter). Chimeras of Nter-truncated Ii (IiΔN, G34-M233) or Cter-truncated CD4 (CD4ΔC, M1-C422) and intracellular domains of CALHM1 or 3 with an additional start methionine as necessary (Fig. 4A) were created by InFusion HD PCR cloning technology (Clontech). For untagged protein expression, coding sequences were cloned in pcDNA3.1(−) (Thermo Fisher). For carboxyl-terminal fusion of a protein to EGFP or 3 × FLAG, its coding sequence without a stop codon was cloned in-frame into pEGFP-N1 (Clontech) or p3 × FLAG^3^. Point mutations involved site-directed mutagenesis.

### Cell culture

The Madin-Darby canine kidney II (MDCKII) epithelial cell line was a gift from Dr. Masayuki Murata (Tokyo University). Cells were maintained in Dulbecco’s Modified Eagle’s medium (DMEM) supplemented with 10% fetal bovine serum (FBS) and 1× Antibiotic-Antimycotic (Gibco) at 37 °C in a humidified incubator with 5% CO_2_-in-air. To produce polarized monolayers, cells were seeded on Transwell filter inserts (Corster) at 3 × 10^5^ cells per 3.2-mm filter for immunostaining, or 5 × 10^5^ cells per 24-mm filter for the domain-specific biotinylation assay, and cultured for 3 or 7 days, respectively.

### Transient transfection of polarized MDCKII cells

Culture medium was replaced with DMEM with reduced CaCl_2_ (5 µM) and cells were cultured for another 2 days. They were then washed once and incubated for 15 min in PBS containing 1 mM MgCl_2_, before being incubated with a transfection mixture of expression vectors and Lipofectamine 3000 in OPTI-MEM (Invitrogen) on both apical (80 µL) and basolateral (20 µL) sides at 37 °C. Expression vectors of CALHM1 fused with EGFP (CALHM1-GFP, 0.5 µg) and/or untagged CALHM3 (1.5 µg) were transfected except that 1.5 µg of untagged CALHM1 and 0.5 µg of CALHM3 fused to EGFP (CALHM3-GFP) were transfected (Fig. 2F). Normal DMEM was added to both sides 5–7 h later (80 and 400 µL to apical and basolateral sides, respectively). Cells underwent immunostaining within 15~20 h post-transfection.

### Immunostaining

Cells were treated with 100 µg/mL cycloheximide (CHX) at 37 °C for 2 h to inhibit protein synthesis and promote visualization of the cell-surface localization of CALHM proteins^1,3^, unless otherwise stated. Cells were washed with ice-cold PBS twice. They were then fixed immediately or subjected to surface biotinylation of the apical membrane before fixation. For apical biotinylation, cells were incubated for 30 min at 4 °C with 0.5 mg/mL EZ-Link sulfo-NHS-SS-biotin (Thermo Scientific) in PBS containing 0.1 mM CaCl_2_ and 1 mM MgCl_2_ (PBS-Ca) on the apical side and PBS-Ca on the basolateral side. The apical biotin solution was refreshed 15 min after the start of incubation. The biotinylation reaction was stopped by adding glycine to the final concentration of 100 mM and two washes with ice-cold PBS containing 100 mM glycine. After the final wash, cells were fixed with 4% paraformaldehyde in PBS for 20 min, permeabilized with 0.25% Triton X-100 in PBS for 30 min, and blocked in PBS containing 5% normal goat serum (PBS-NGS) for 1 h (all at room temperature). They were then incubated at 4 °C overnight with primary antibodies diluted in PBS-NGS: mouse anti-GFP (Clontech), rabbit anti-Na^+^/K^+^ ATPase (Abcam), and rabbit anti-biotin (Abcam) antibodies. The following day, cells were incubated at room temperature for 1 h with Alexa Fluor-conjugated secondary antibodies (Thermo Fisher Scientific) in PBS-NGS, before mounting in Vectashield (Vector Laboratories). Confocal images were acquired using a LSM510 microscope with an EC Plan-Neofluar 100×/1.30 NA Oil objective and the pinhole set to 1 Airy Unit for the red channel and adjusted to yield the same optical slice thickness in the green channel. Images show single optical XY planes or XZ planes reconstructed from Z-stack images, which were taken from the apical to basal ends of cells at depth intervals of half the optical slice thickness and deconvoluted and reconstructed using Image J software (NIH).

### Generation of stable cell lines

MDCKII cells in a 3.5-cm dish were transfected at 50~70% confluency with 2.5 µg of an expression vector using Lipofectamine 3000. The next day, cells were collected by trypsinization and plated in ten 10-cm dishes. Isolated colonies of stably-transfected clones formed in the presence of 800 µg/mL of G418 (Cosmobio) were collected after 10~14 days. Protein expression of transfected genes was confirmed by Western blotting.

### Domain-specific surface biotinylation

Surface biotinylation specific to the apical or basolateral membrane domain of MDCKII epithelia was performed using the same procedure as described in the Immunostaining section above, but the biotin solution was applied to either the apical or basolateral side. After stopping biotinylation by washing with PBS containing 100 mM glycine, cells were washed with ice-cold PBS twice, harvested in 70 µL of lysis buffer (PBS containing 1% Triton X-100, 1 mM phenylmethylsulfonyl fluoride, and 1× protease inhibitor cocktail (Sigma-Aldrich)), and centrifuged at 15,000 × g and 4 °C for 10 min. Supernatants were collected as whole-cell lysates. Biotinylated proteins in 300 µg of the whole-cell lysates were pulled down by 50 µL of NeutrAvidin agarose resin (Thermo Fisher Scientific) and eluted by incubating the resin in 50 µL of Laemmli sample buffer at 37 °C for 1 h. The whole-cell lysates (100 µg) were denatured in Laemmli sample buffer (Total), and the total amount of the eluate samples (Surface) was subjected to SDS-PAGE/Western blotting^3^. Primary antibodies were mouse anti-FLAG (Sigma), rabbit anti-Na^+^/K^+^ ATPase (Abcam), and mouse anti-β-tubulin (Thermo Fisher). The apical membrane distribution of a 3 × FLAG-tagged protein expressed in the entire plasma membrane is presented as a percentage: 100 × (I_Api.Surf_/I_Api.Tot_)/[(I_Api.Surf_/I_Api.Tot_) + (I_Baso.Surf_/I_Baso.Tot_)] (%), where I_Api.Surf_ and I_Api.Tot_ are band intensities of a target protein in the eluate and whole-cell lysate of apically-biotinylated cells, respectively, and I_Baso.Surf_ and I_Baso.Tot_ are those of basolaterally-biotinylated cells. The Na^+^/K^+^ ATPase localization restricted to the basolateral membrane (>90%) was used as a criterion for successful polarization of the epithelia.

### Statistical analyses

Data are means ± SEM. Comparison involved one-way analysis of variance followed by Bonferroni post-hoc tests. *P*-values < 0.05 were significant. N = number of experiments.

## Supplementary information

Supplementary Information

## Data Availability

The datasets generated during and/or analysed during the current study are available from the corresponding author on reasonable request.
