# Supplementary material for: CALHM1/CALHM3 channel is intrinsically sorted to the basolateral membrane of epithelial cells including taste cells
Source: Sci Rep. 2019 Feb 25;9:2681. doi: 10.1038/s41598-019-39593-5 (PMC6390109; doi:10.1038/s41598-019-39593-5)

CALHM1/CALHM3 channel is intrinsically sorted to the basolateral membrane of epithelial cells including taste cells

Makiko Kashio[1,#], Gao Wei-qi[3], Yasuyoshi Ohsaki[3], Mizuho A. Kido[3], Akiyuki Taruno[1,2,*]

[1]Department of Molecular Cell Physiology, Kyoto Prefectural University of Medicine, Kyoto 602-8566, Japan.
[2]JST, PRESTO, Kawaguchi, Saitama, 332-0012, Japan
[3]Department of Anatomy and Physiology, Saga University, Saga 849-8501, Japan.

[*]Correspondence to:
Akiyuki Taruno, M.D., Ph.D.
Department of Molecular Cell Physiology
Kyoto Prefectural University of Medicine
465 Kajiicho Kamigyo-ward, Kyoto 602-8566, Japan
Tel: +81-75-251-5310
Fax: +81-75-251-0295
Email: taruno@koto.kpu-m.ac.jp

[#]Present address: Department of Physiology, Aichi Medical University, Nagakute 480-1195, Japan

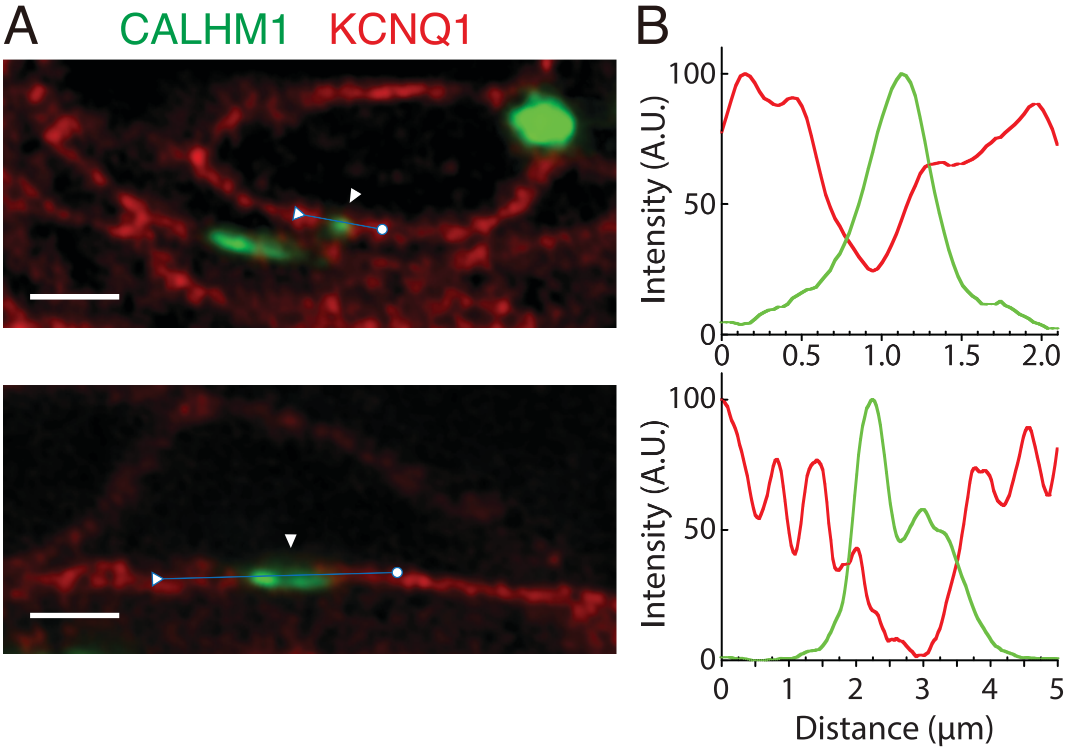

Supplementary figure 1

Localization of CALHM1 in the basolateral membrane of TBCs.

(A) Airyscan images of TBCs in the circumvallate taste buds double-stained for CALHM1 (green) and KCNQ1 (red). Scale bar, 2 μm. Data are representative of seven Airyscan images. **(B)** Line plots of basolateral membrane areas carrying a single CALHM1 punctum shown in (A). CALHM1 signal, green line; KCNQ1 signal, red line; A.U., arbitrary units.

Supplementary figure 2.
Uncropped blots for images shown in Fig. 5B

CD4ΔC-CAL1 Cter (FLAG)

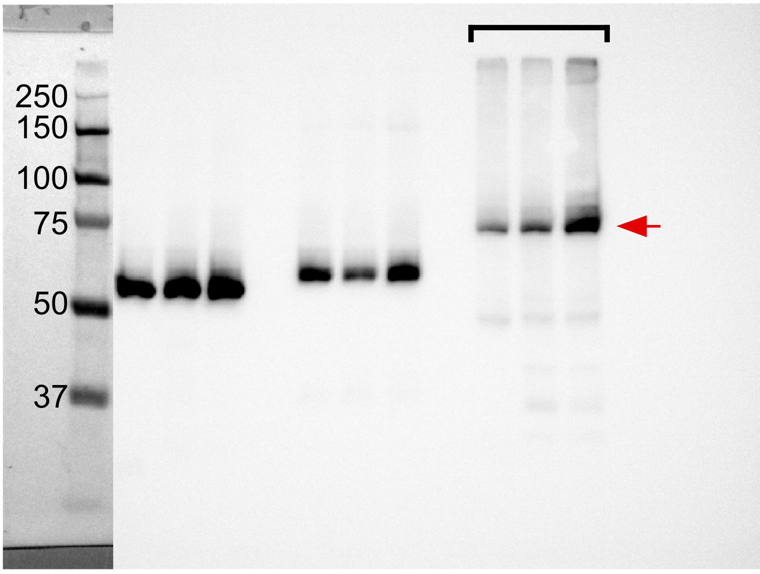

CD4ΔC-CAL1 Cter (β-tubulin)

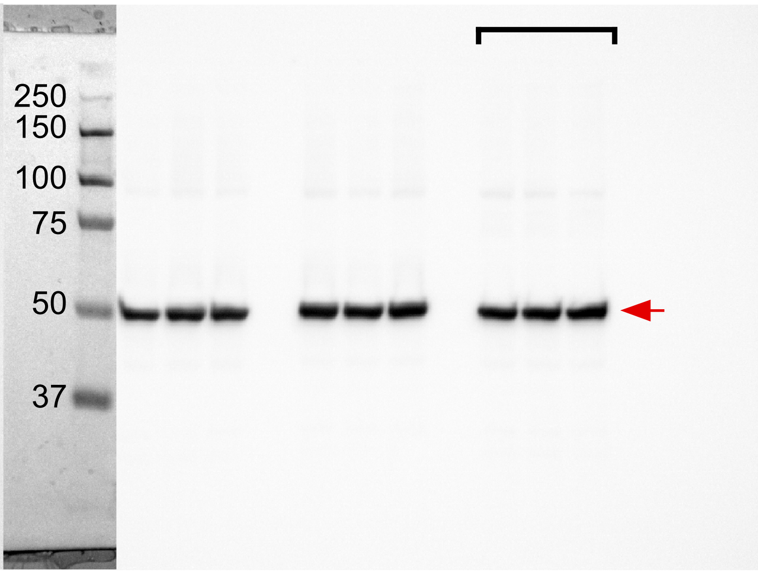

CD4ΔC-CAL3 Cter (FLAG)

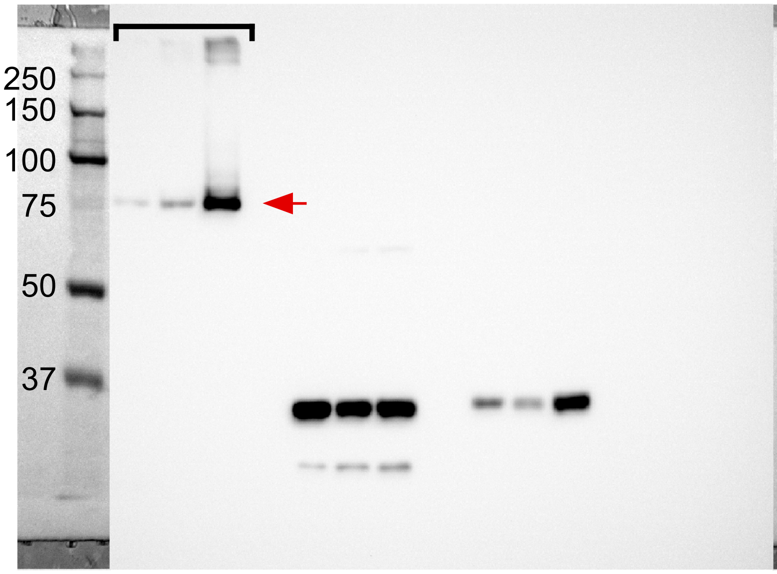

CD4ΔC-CAL3 Cter (β-tubulin)

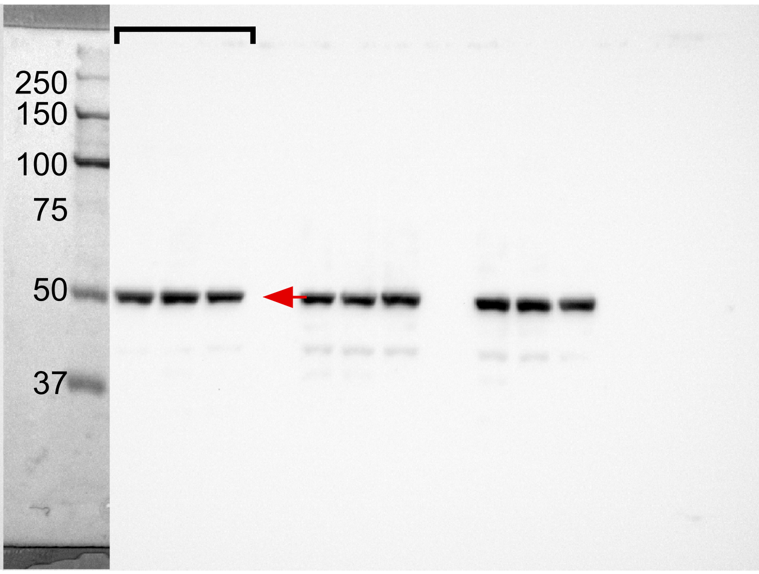

Supplementary figure 3
Uncropped blots for images shown in Fig. 5C

IiΔN (FLAG), Input (short exposure)

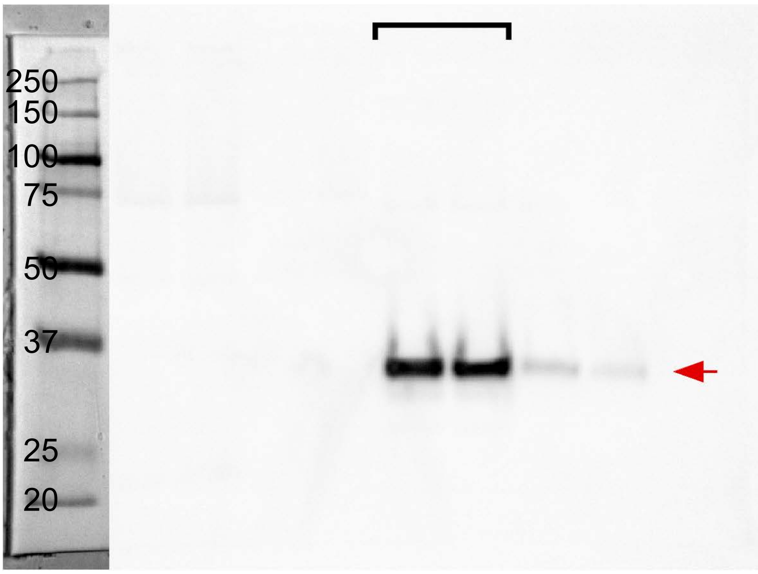

IiΔN (FLAG), Surface (long exposure)

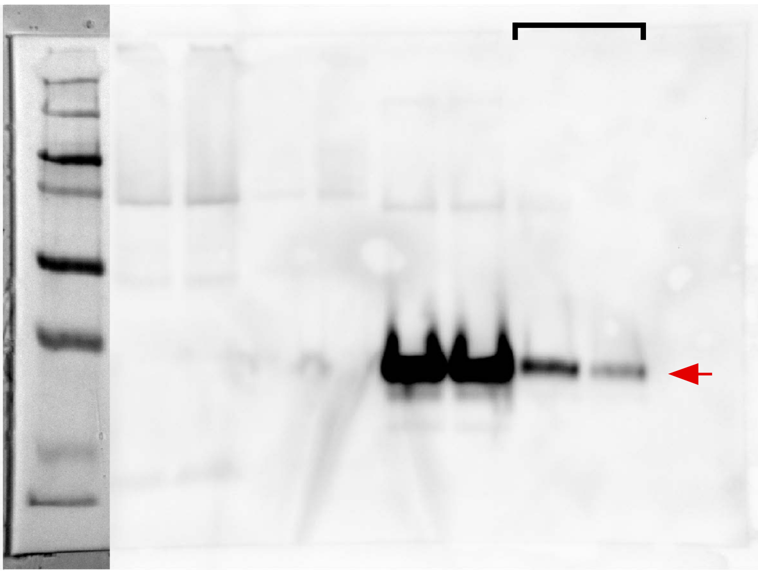

IiΔN (Na/K ATPase)

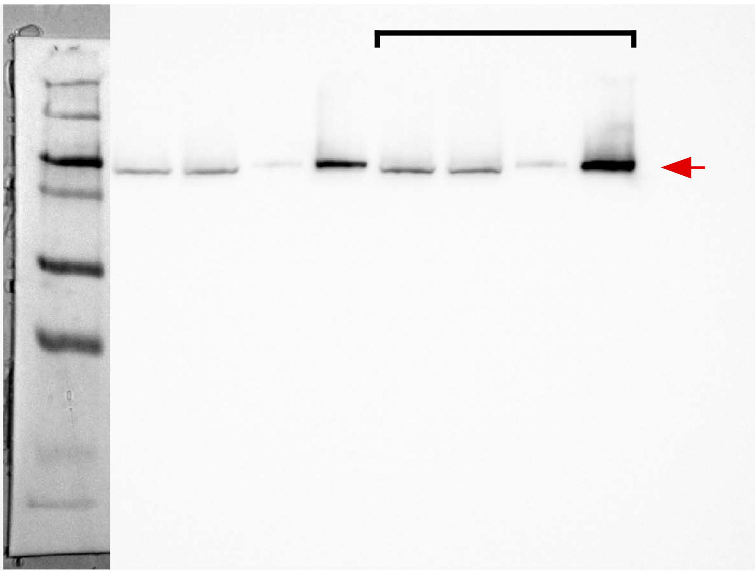

IiΔN (β-tubulin)

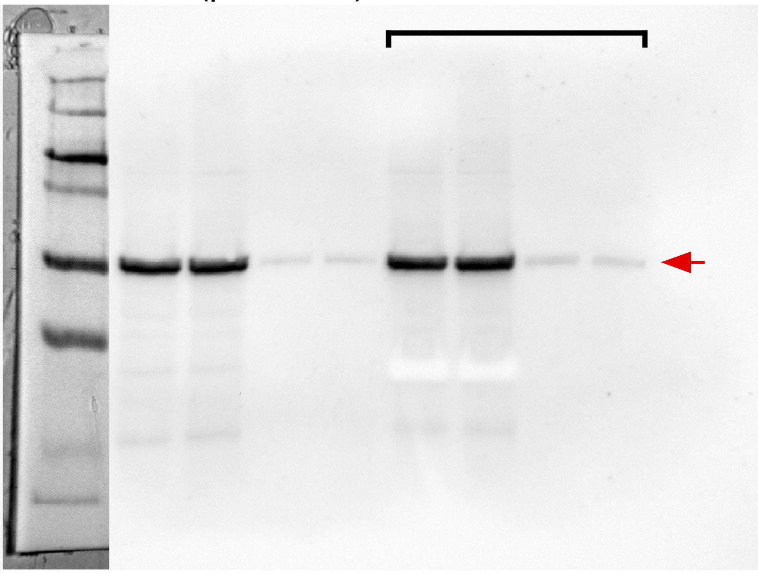

Supplementary figure 3
Uncropped blots for images shown in Fig. 5C

CALHM1 Nter-IiΔN (FLAG),
Input (short exposure)

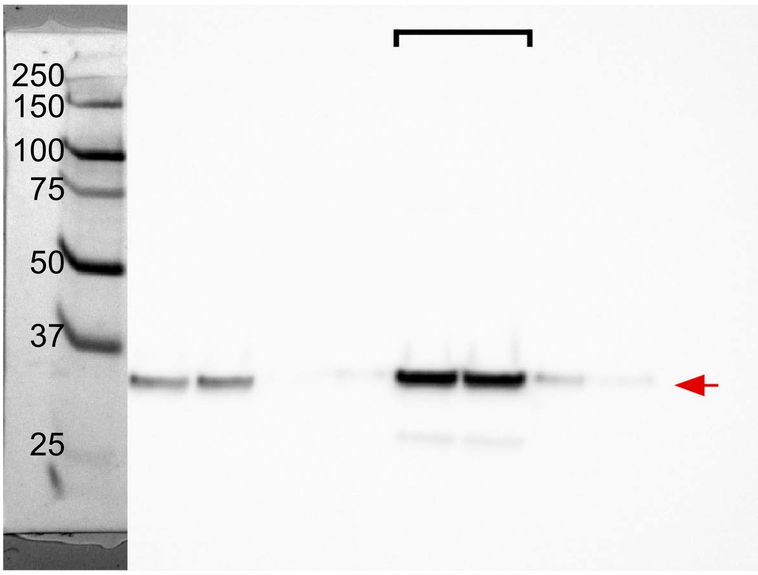

CALHM1 Nter-IiΔN (FLAG),
Surface (long exposure)

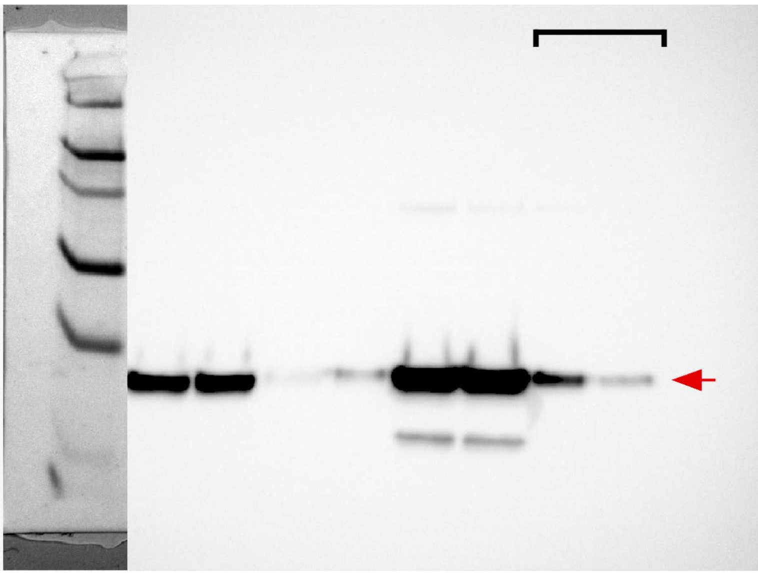

CALHM1 Nter-IiΔN (Na/K ATPase)

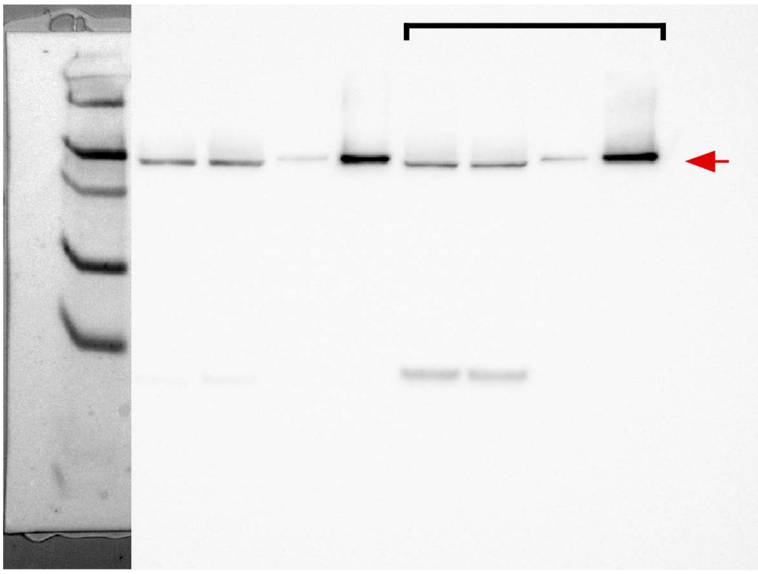

CALHM1 Nter-IiΔN (β-tubulin)

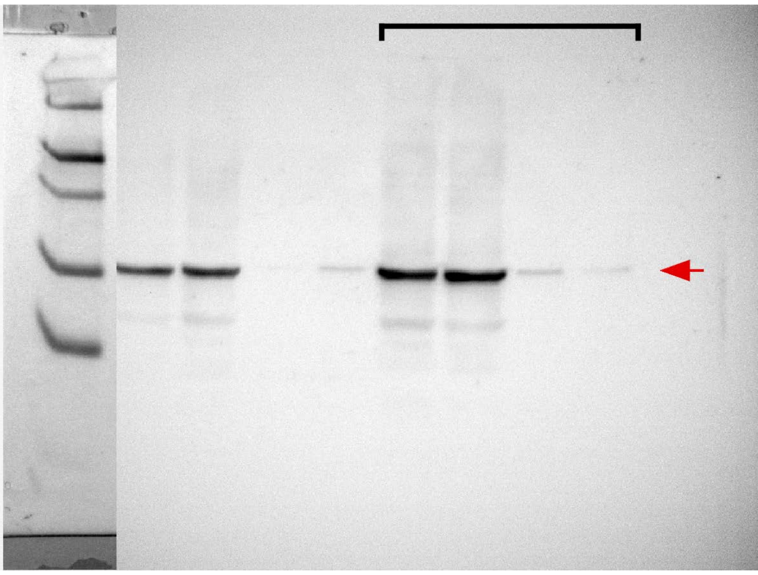

Supplementary figure 3
Uncropped blots for images shown in Fig. 5C

CALHM1 Loop-IiΔN (FLAG),
Input (short exposure)

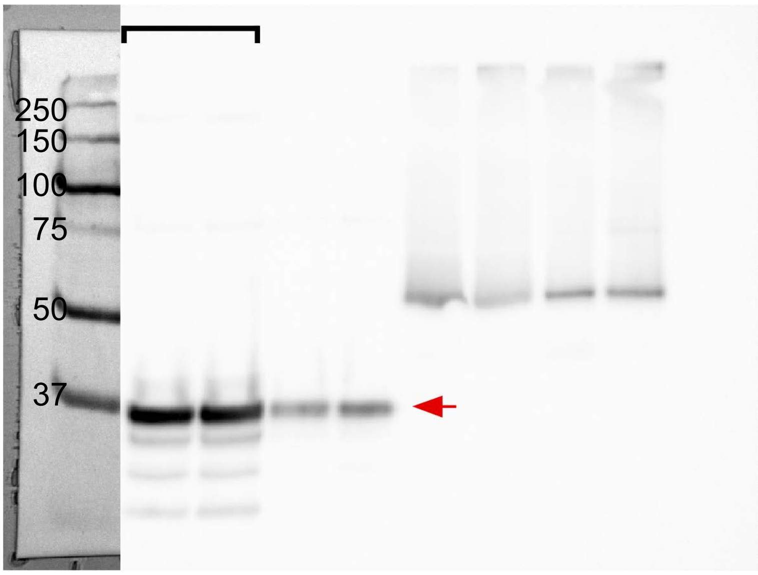

CALHM1 Loop-IiΔN (FLAG),
Surface (long exposure)

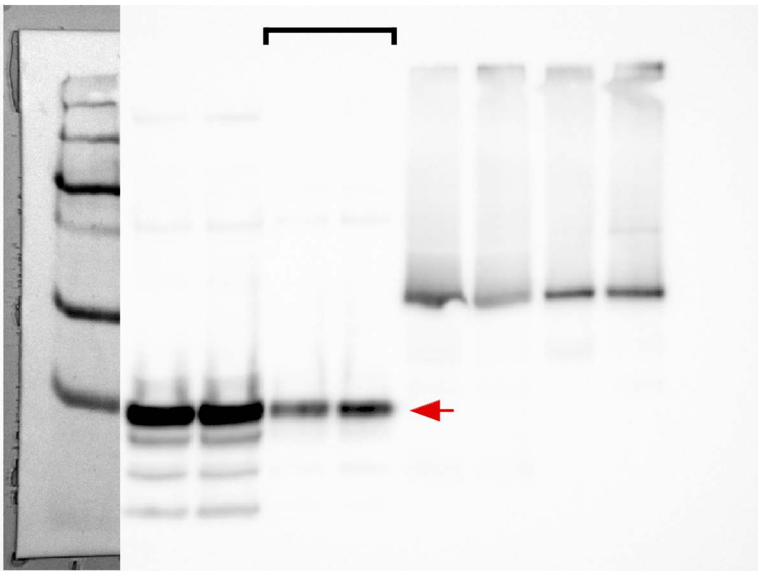

CALHM1 Loop-IiΔN (Na/K ATPase)

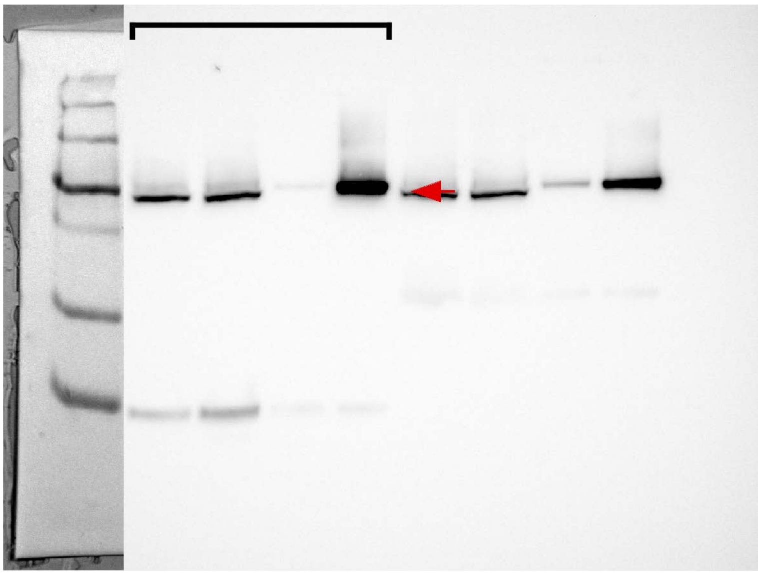

CALHM1 Loop-IiΔN (β-tubulin)

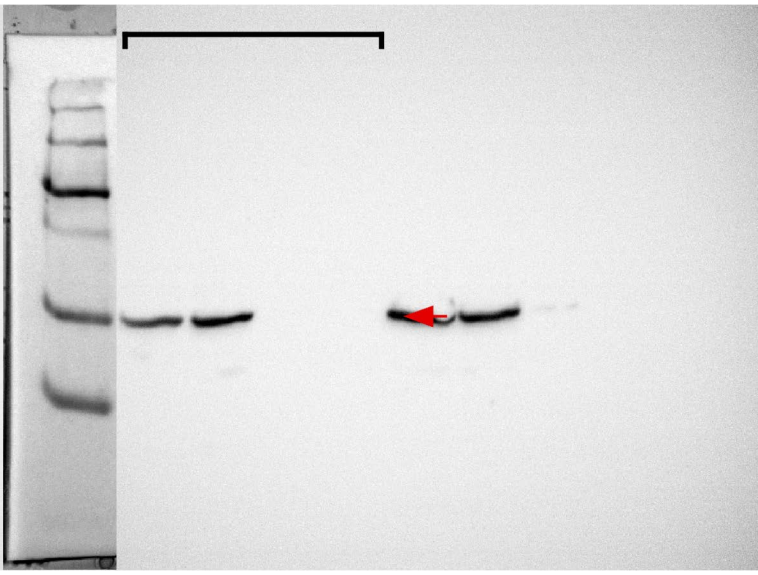

Supplementary figure 3
Uncropped blots for images shown in Fig. 5C

CALHM3 Nterm-IiΔN (FLAG),
Input (short exposure)

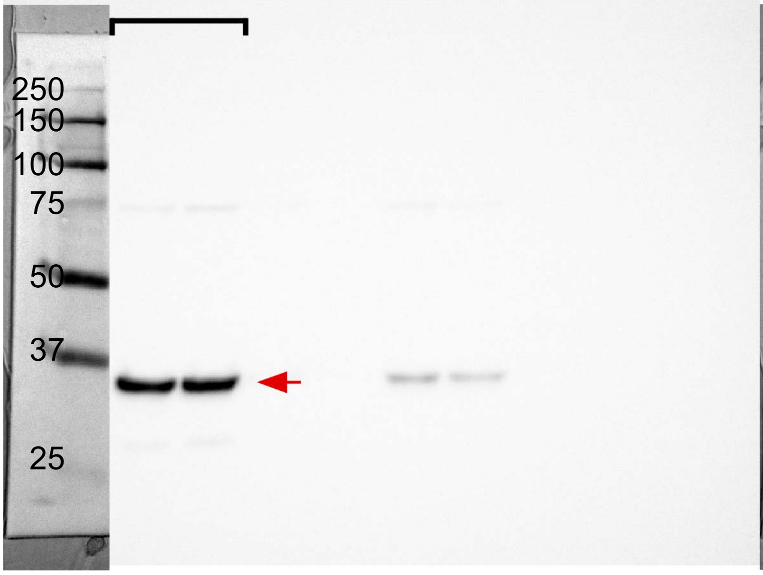

CALHM3 Nterm-IiΔN (FLAG),
Surface (long exposure)

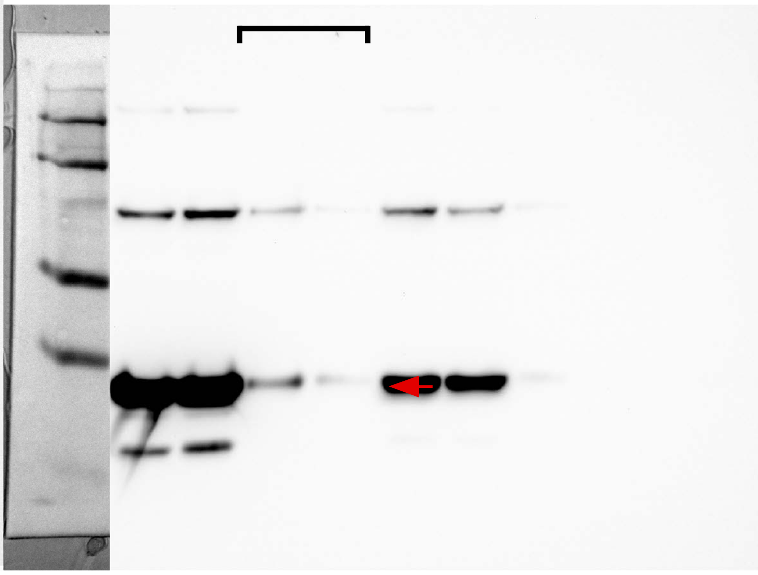

CALHM3 Nterm-IiΔN (Na/K ATPase)

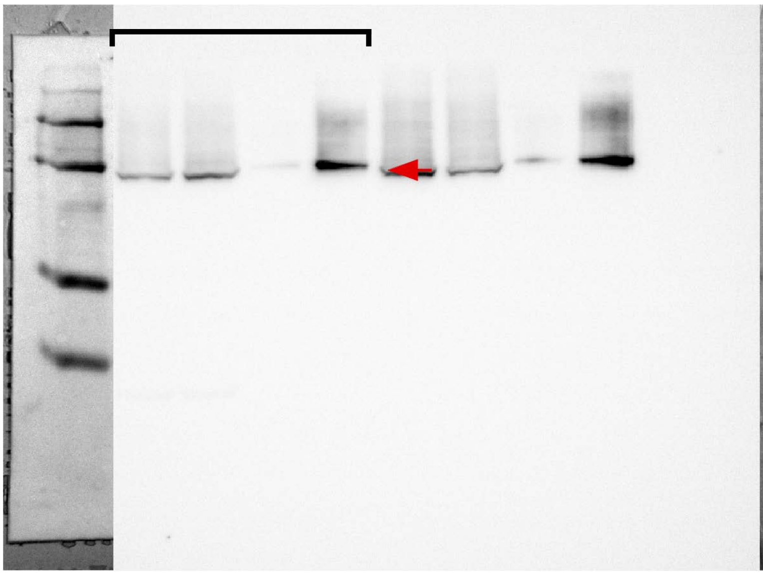

CALHM3 Nterm-IiΔN (β-tubulin)

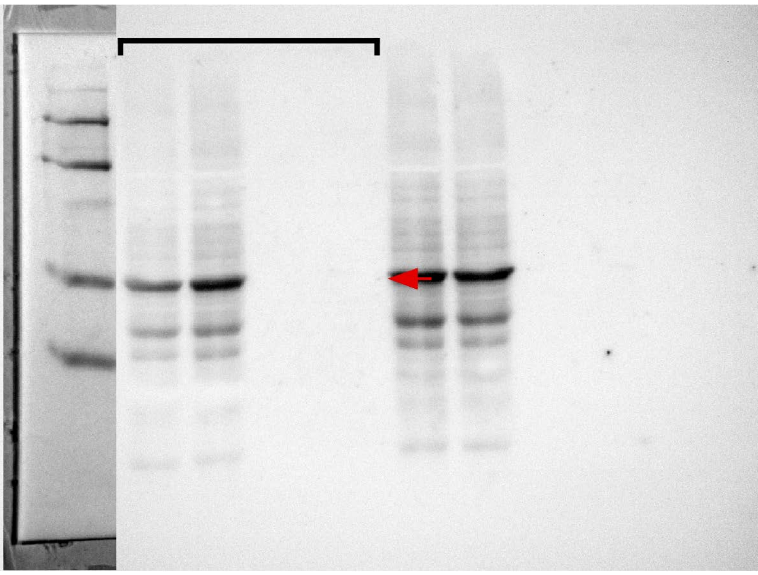

Supplementary figure 3
Uncropped blots for images shown in Fig. 5C

CALHM3 Loop-IiΔN (FLAG),
Input (short exposure)

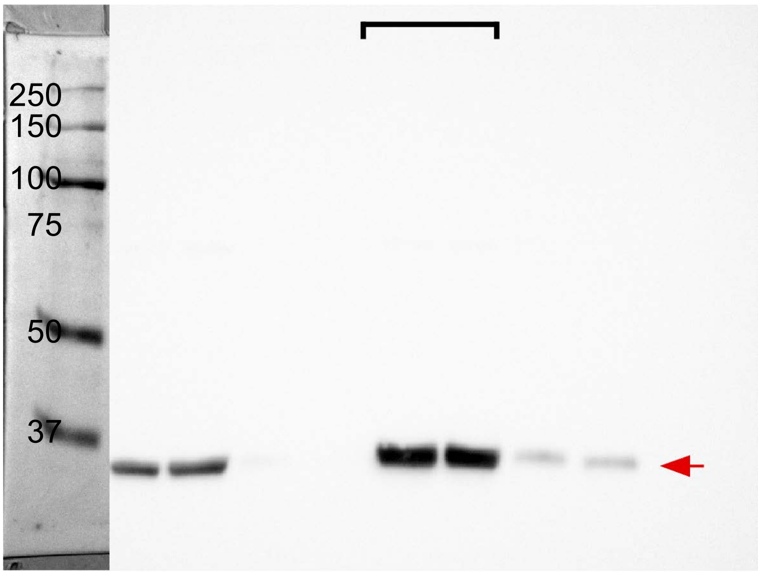

CALHM3 Loop-IiΔN (FLAG),
Surface (long exposure)

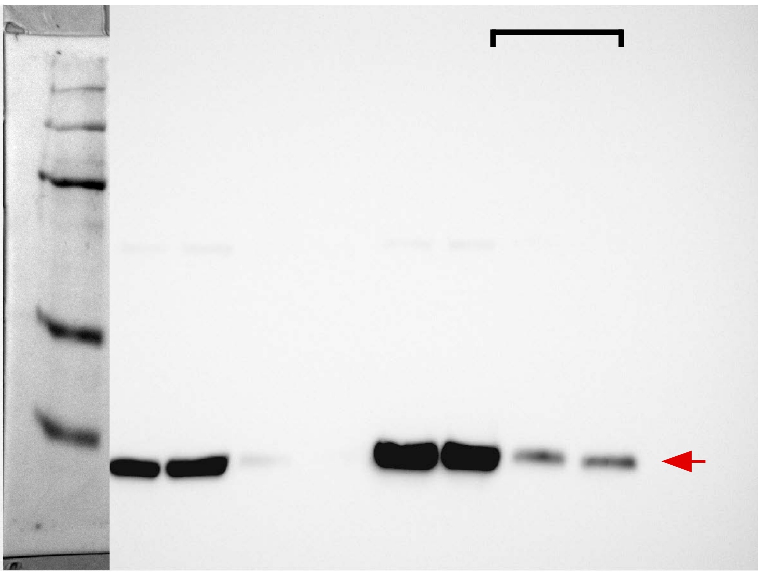

CALHM3 Loop-IiΔN (Na/K ATPase)

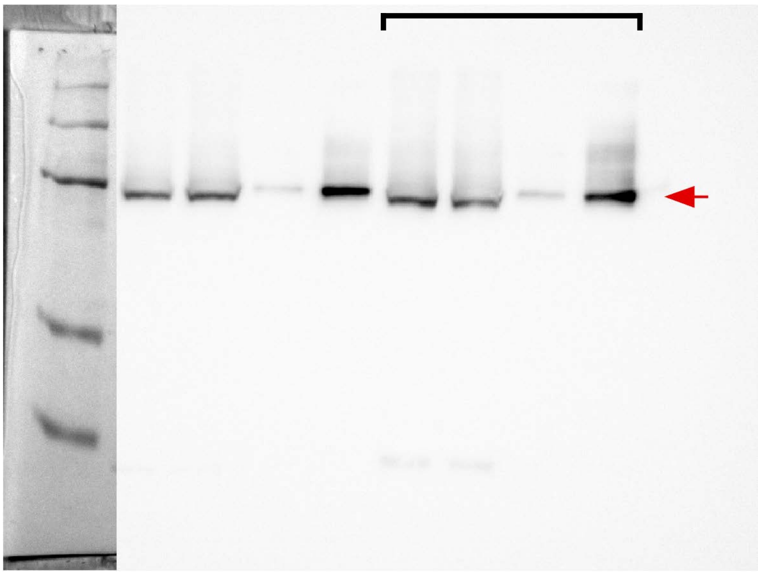

CALHM3 Loop-IiΔN (β-tubulin)

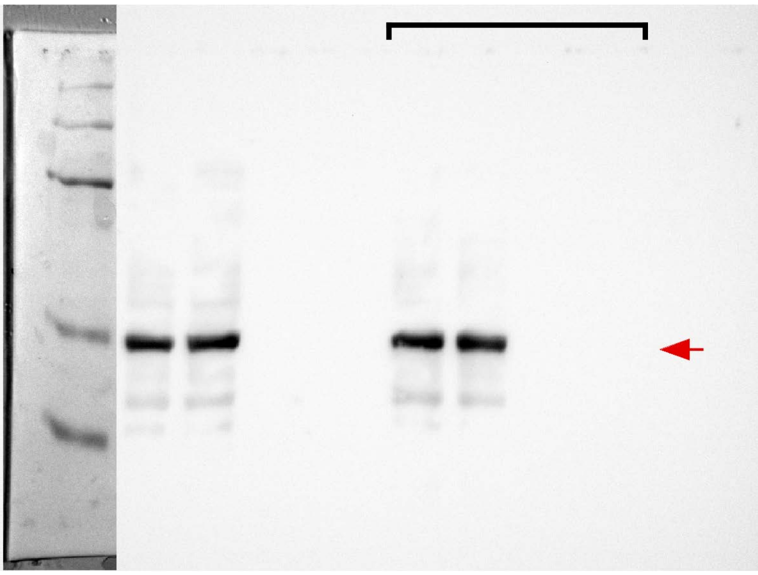

Supplementary figure 3
Uncropped blots for images shown in Fig. 5C

CD4ΔC (FLAG)

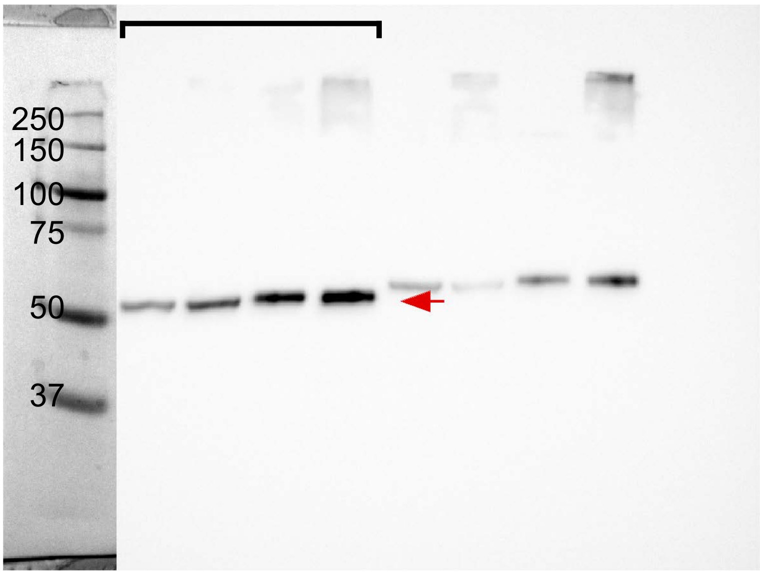

CD4ΔC (Na/K ATPase)

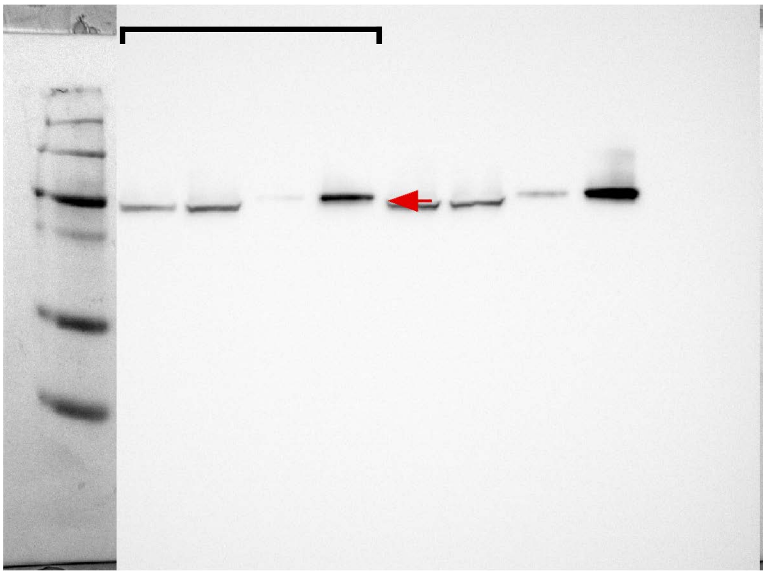

CD4ΔC (β-tubulin)

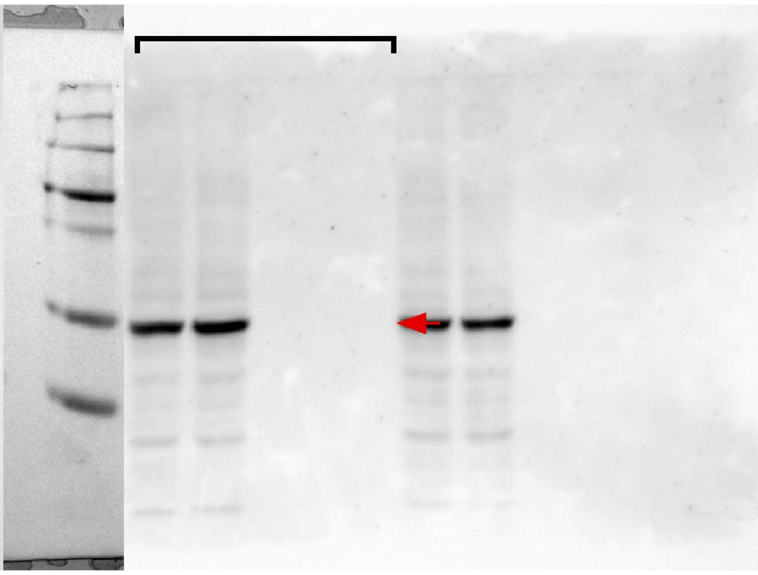

Supplementary figure 3
Uncropped blots for images shown in Fig. 5C

CD4ΔC-CALHM1 Loop (FLAG)

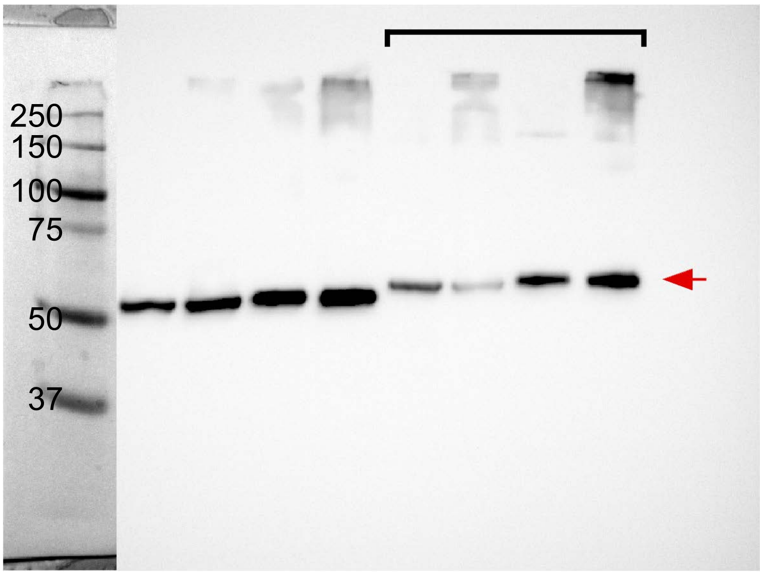

CD4ΔC-CALHM1 Loop (Na/K ATPase)

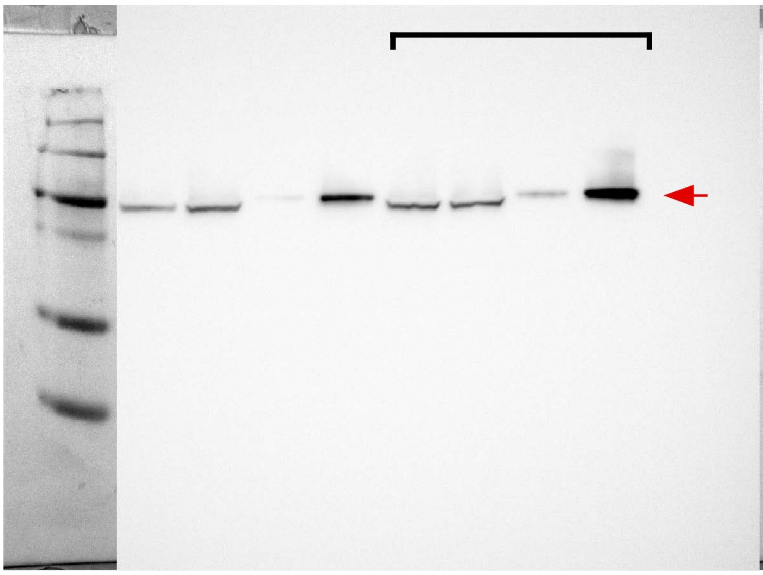

CD4ΔC-CALHM1 Loop (β-tubulin)

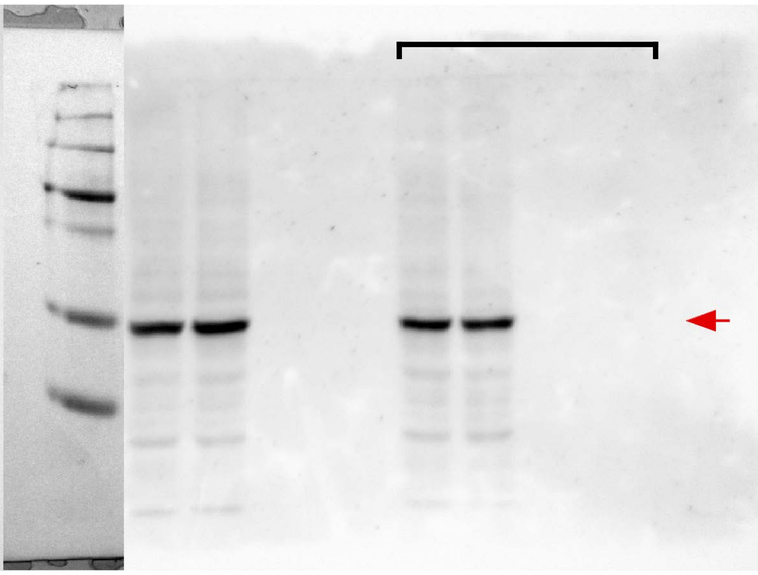

Supplementary figure 3
Uncropped blots for images shown in Fig. 5C

CD4ΔC-CALHM3 Loop (FLAG),
Surface (short exposure)

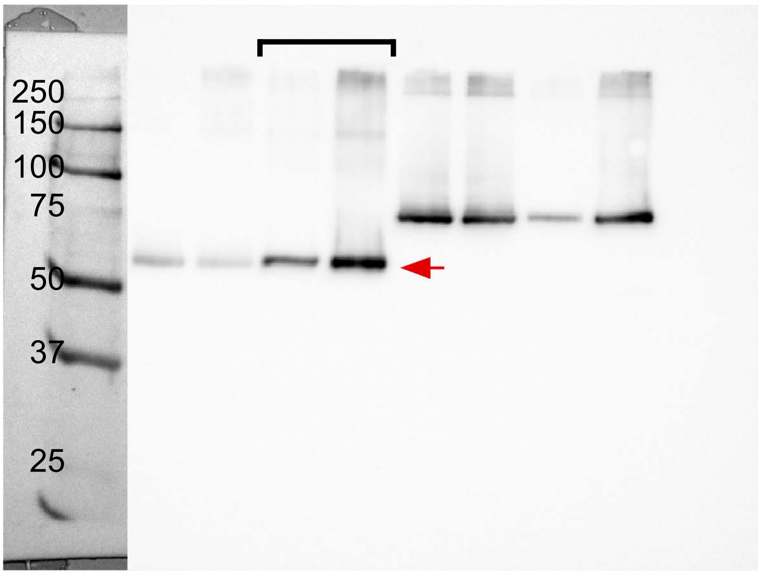

CD4ΔC-CALHM3 Loop (FLAG),
Input (long exposure)

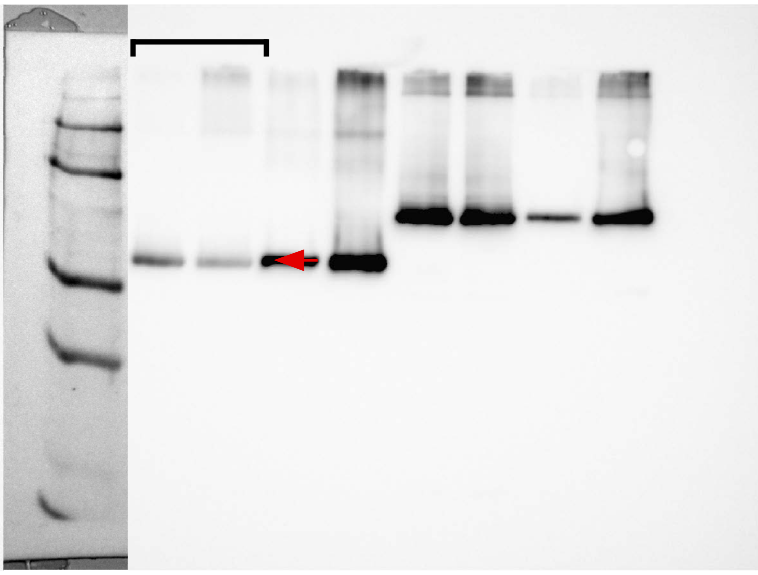

CD4ΔC-CALHM3 Loop (Na/K ATPase)

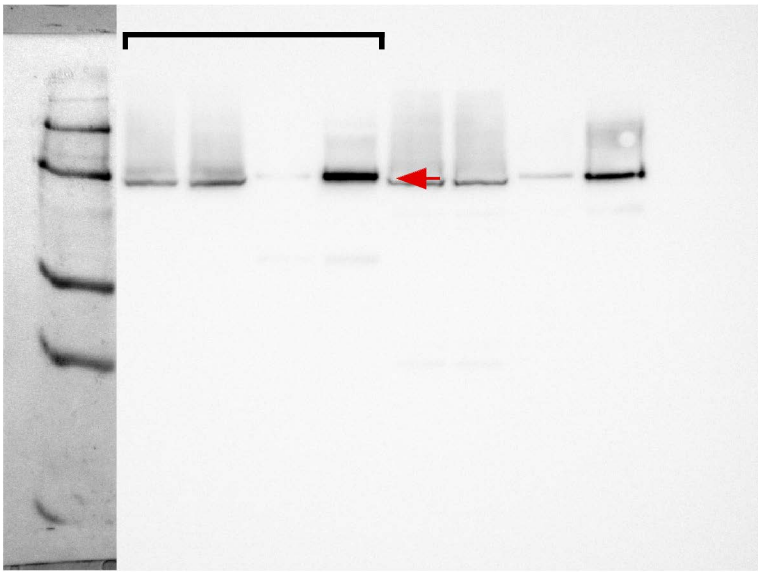

CD4ΔC-CALHM3 Loop (β-tubulin)

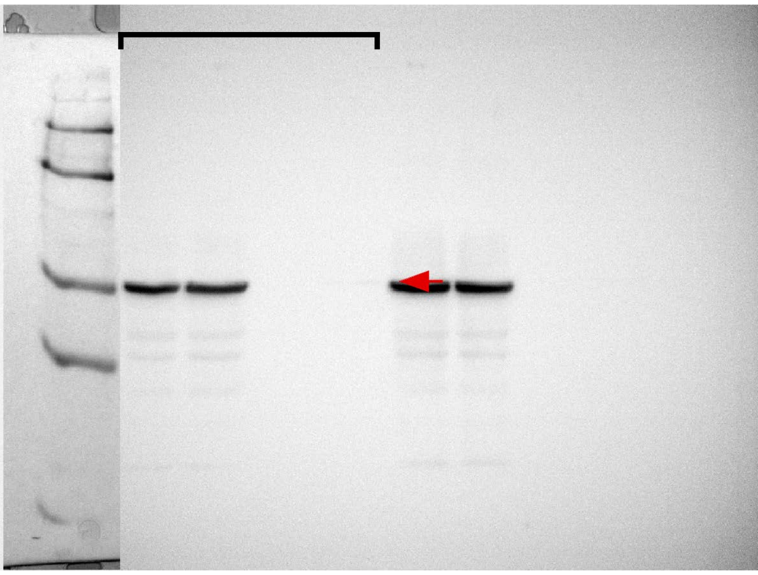

Supplementary figure 3
Uncropped blots for images shown in Fig. 5C

CD4ΔC (+MG) (FLAG)

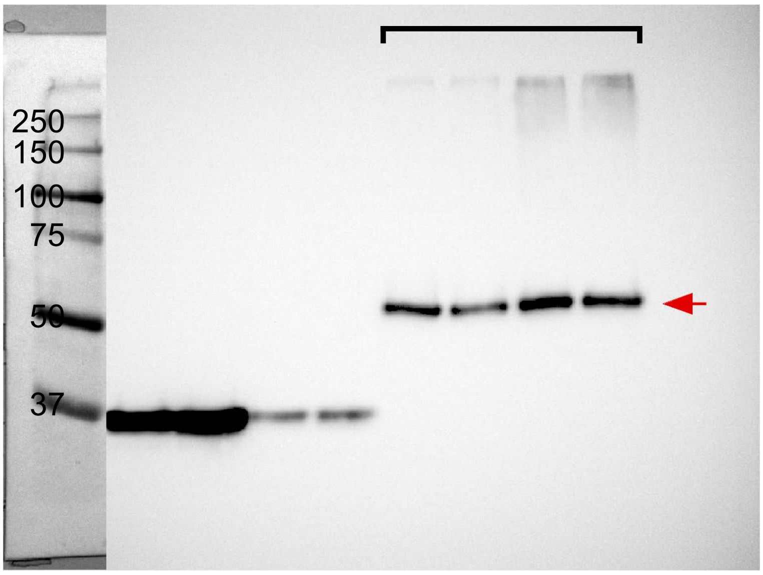

CD4ΔC (+MG) (Na/K ATPase)

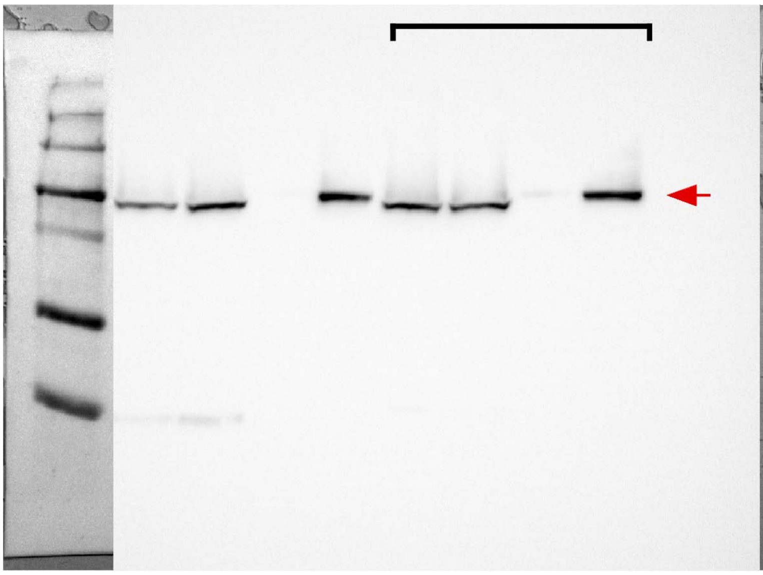

CD4ΔC (+MG) (β-tubulin)

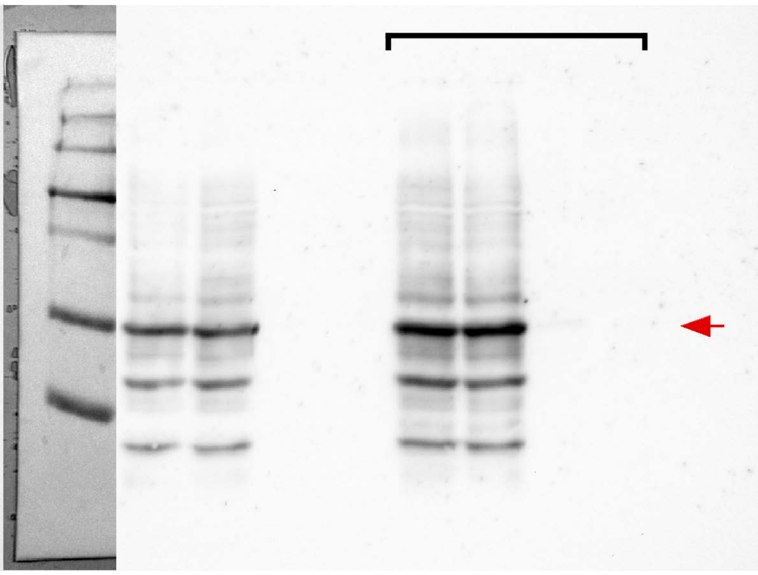

Supplementary figure 3
Uncropped blots for images shown in Fig. 5C

CD4ΔC-CALHM1 Cterm (+MG)
(FLAG)

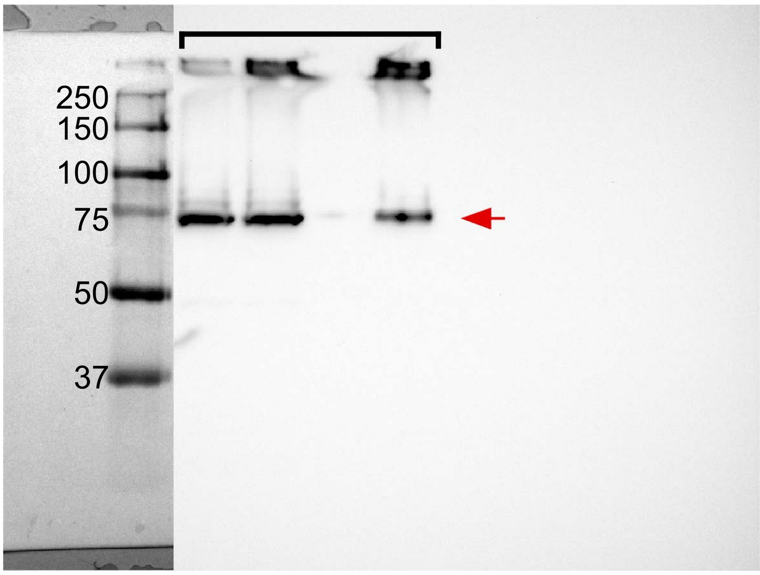

CD4ΔC-CALHM1 Cterm (+MG)
(Na/K ATPase)

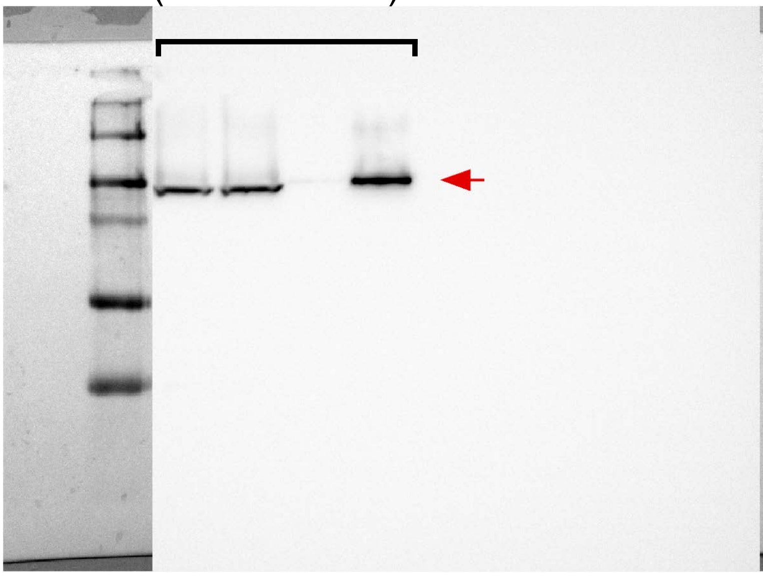

CD4ΔC-CALHM1 Cterm (+MG)
(β-tubulin)

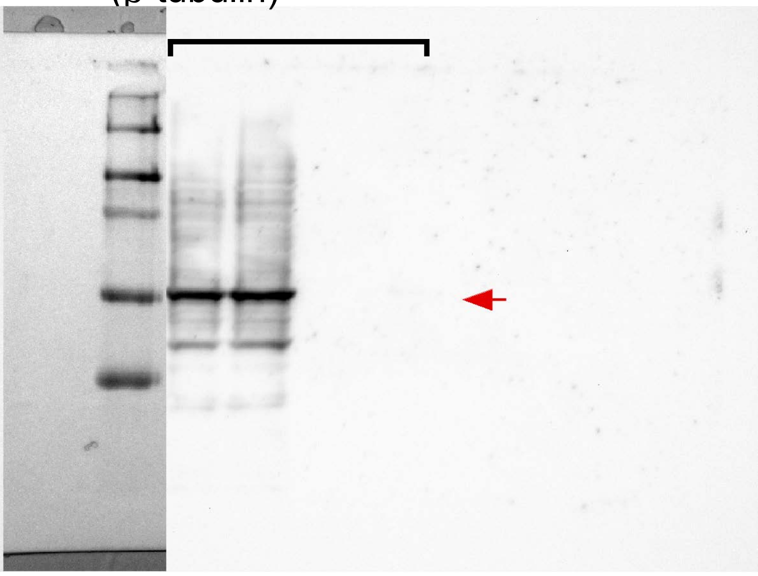

Supplementary figure 3
Uncropped blots for images shown in Fig. 5C

CD4ΔC-CALHM3 Cterm (+MG)
(FLAG)

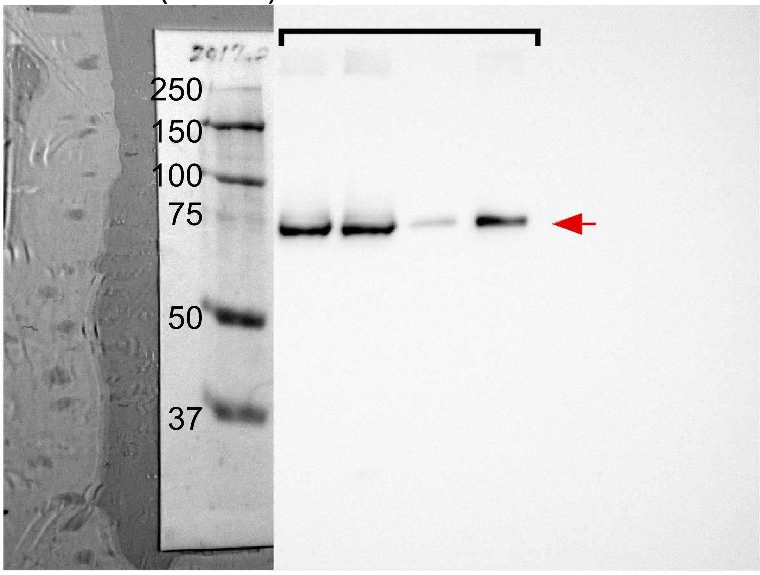

CD4ΔC-CALHM3 Cterm (+MG)
(Na/K ATPase)

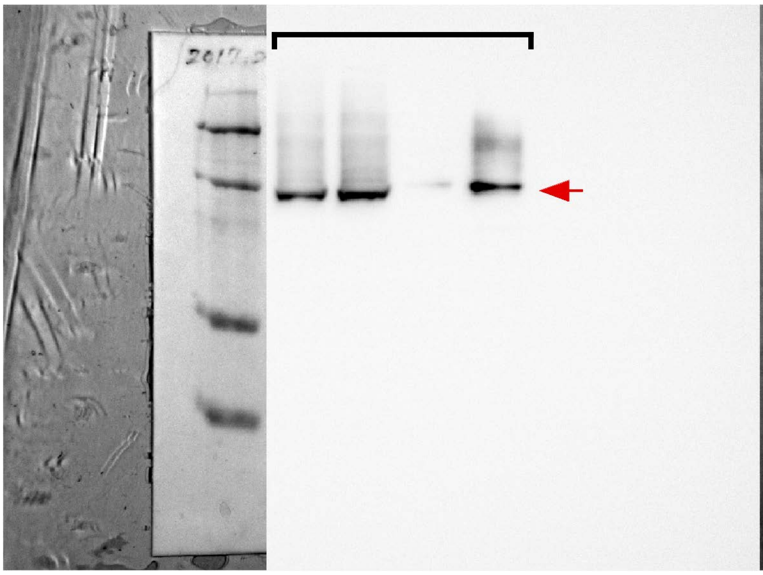

CD4ΔC-CALHM3 Cterm (+MG)
(β-tubulin)

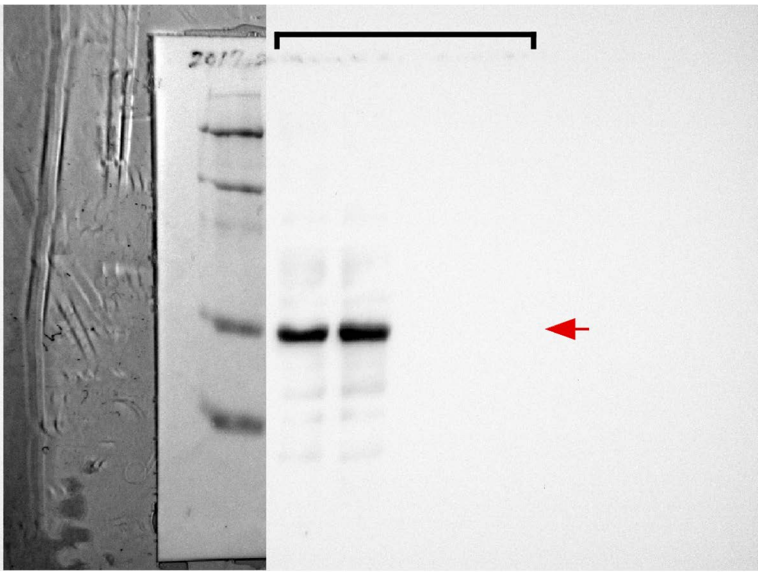

Supplementary figure 4
Uncropped blots for images shown in Fig. 7A

CD4ΔC (FLAG)

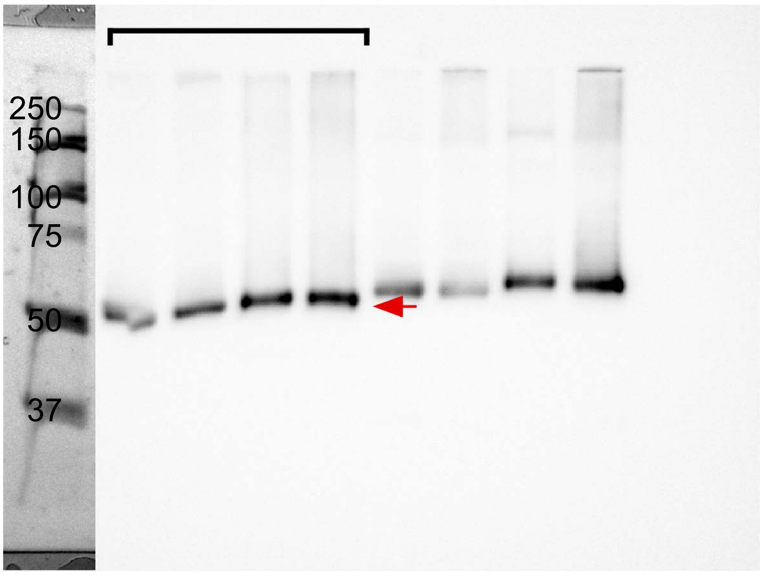

CD4ΔC (Na/K ATPase)

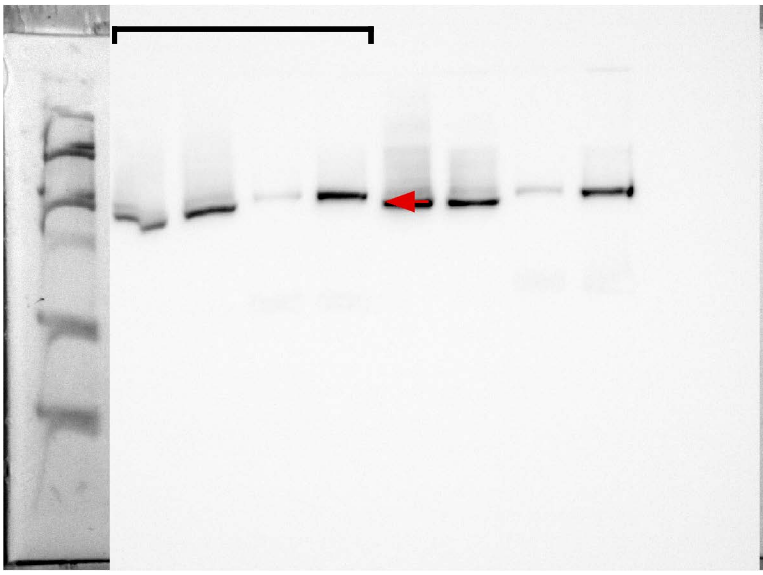

CD4ΔC (β-tubulin)

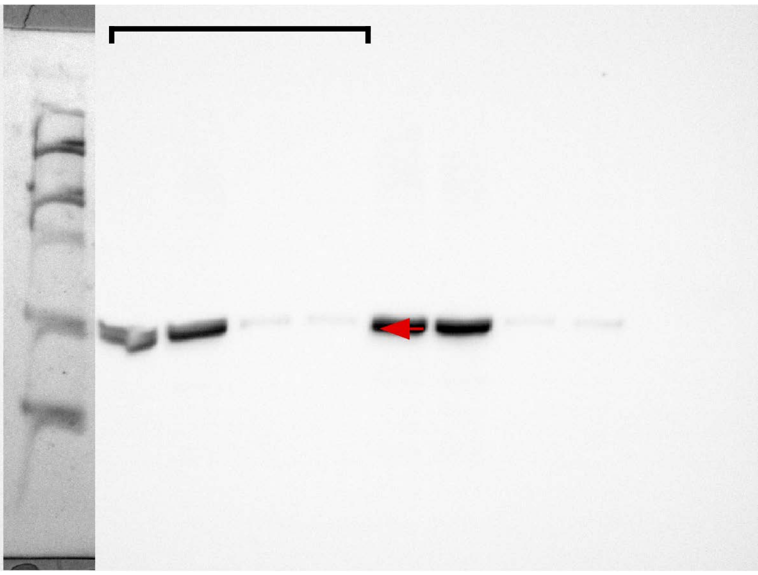

Supplementary figure 4
Uncropped blots for images shown in Fig. 7A

CD4ΔC-CALHM1 Loop (FLAG)

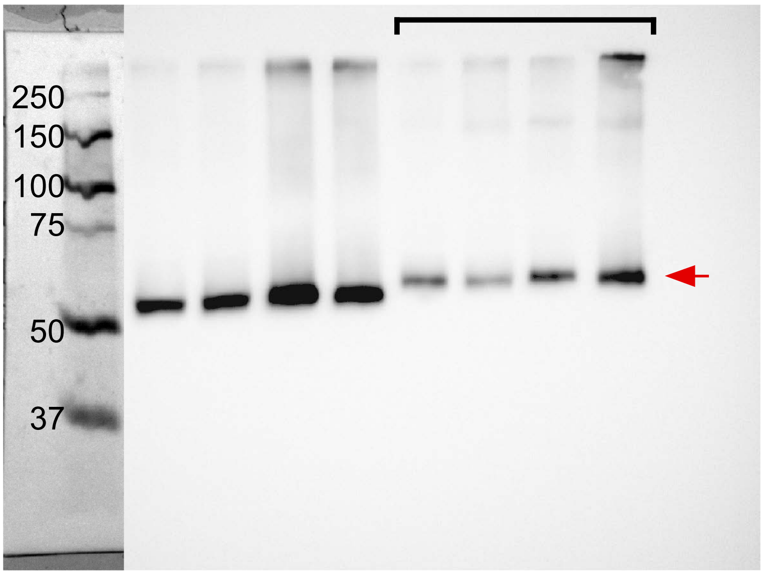

CD4ΔC-CALHM1 Loop (Na/K ATPase)

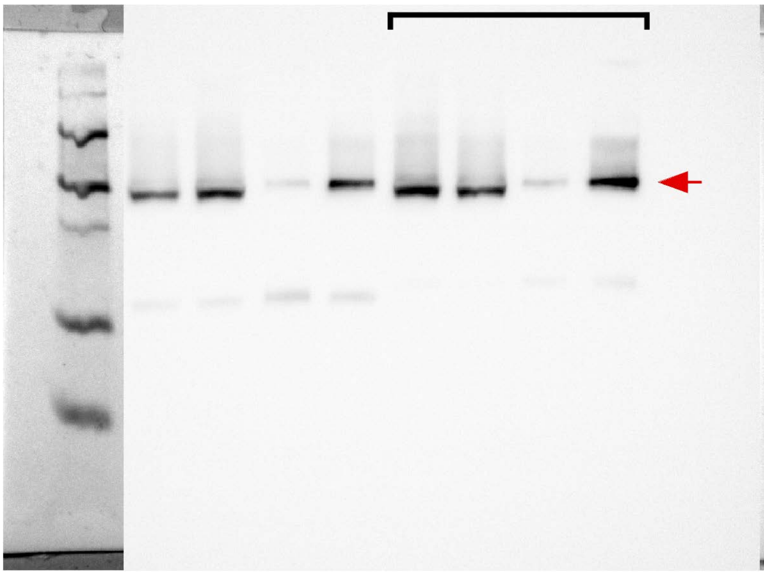

CD4ΔC-CALHM1 Loop (β-tubulin)

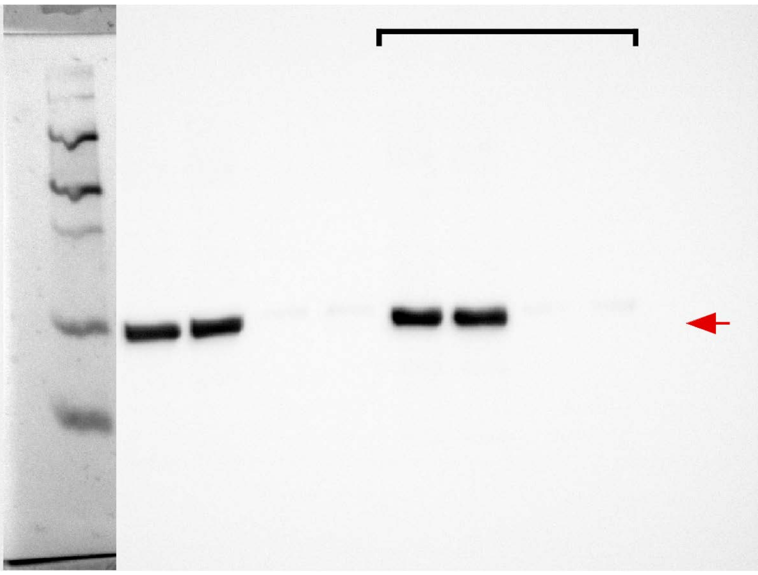

Supplementary figure 4
Uncropped blots for images shown in Fig. 7A

CD4ΔC-CALHM1 Loop (ML/AA) (FLAG)

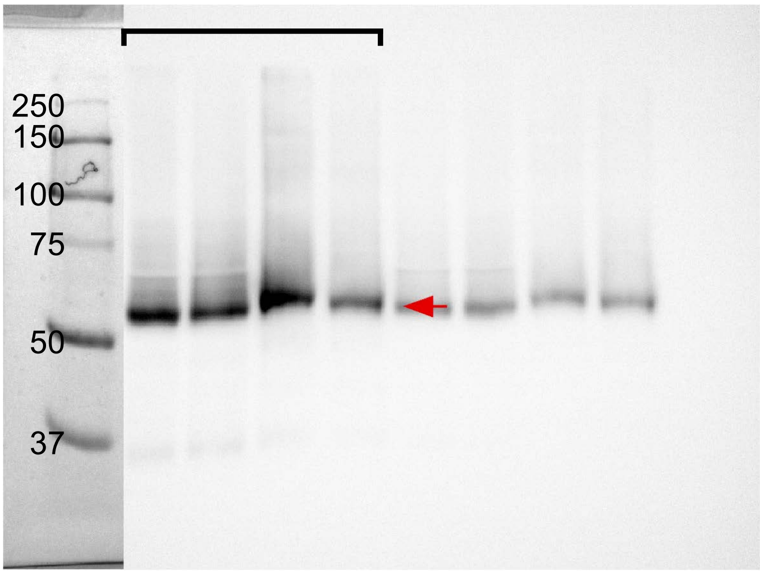

CD4ΔC-CALHM1 Loop (ML/AA)
(Na/K ATPase)

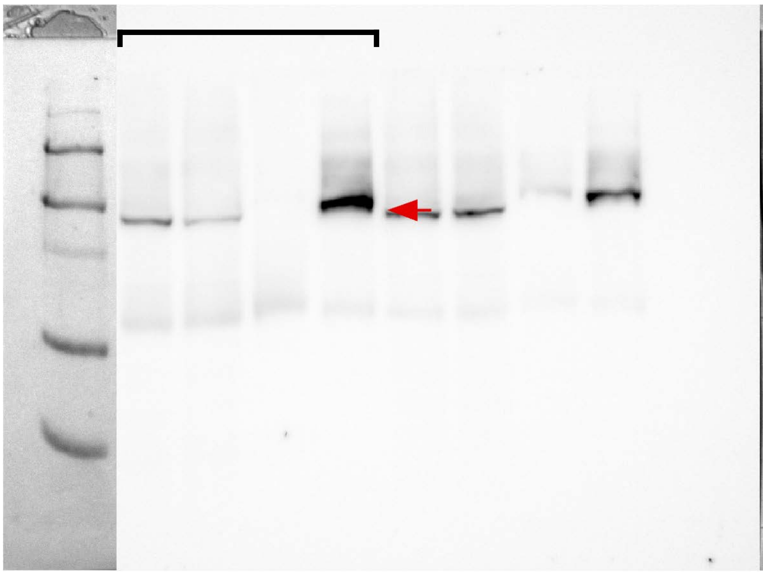

CD4ΔC-CALHM1 Loop (ML/AA)]
(β-tubulin)

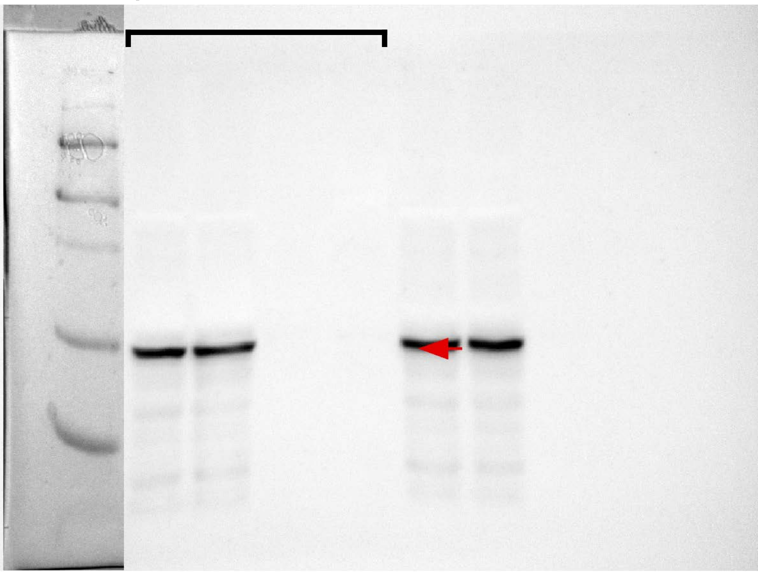

Supplementary figure 4
Uncropped blots for images shown in Fig. 7A

CD4ΔC (+MG) (FLAG)

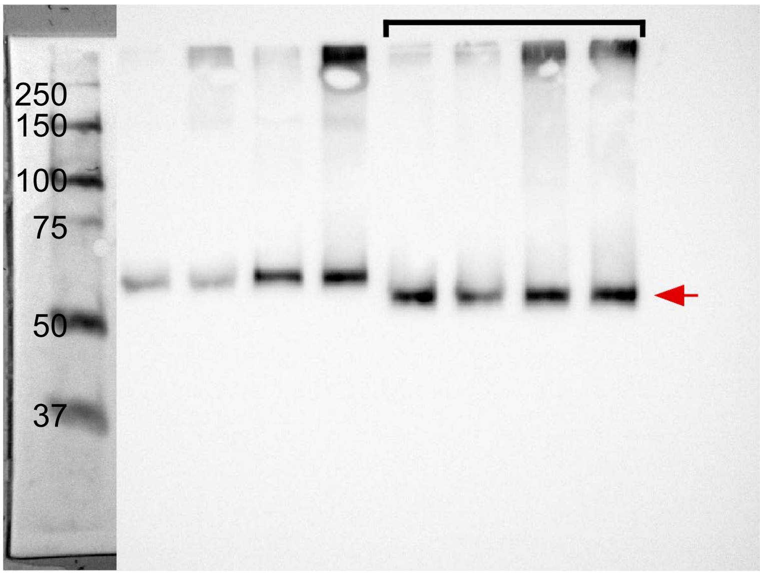

CD4ΔC (+MG) (Na/K ATPase)

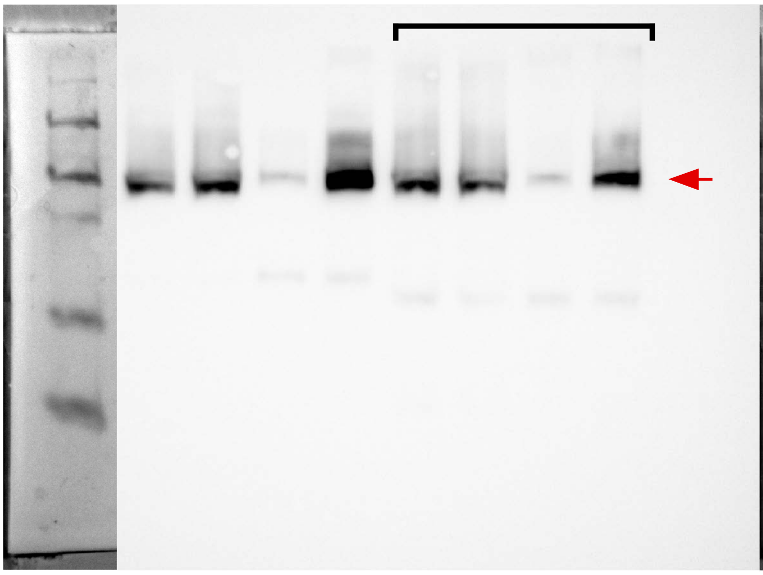

CD4ΔC (+MG) (β-tubulin)

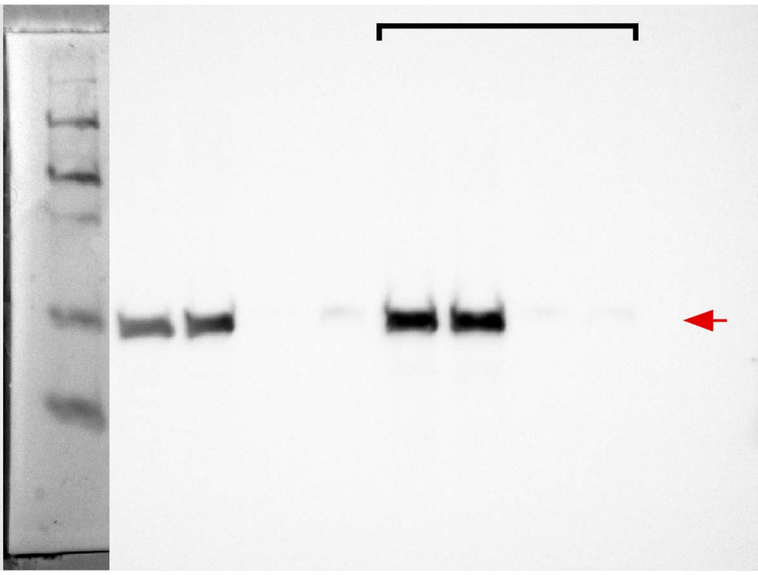

Supplementary figure 4
Uncropped blots for images shown in Fig. 7A

CD4ΔC-CALHM1 Cterm (+MG) (FLAG)

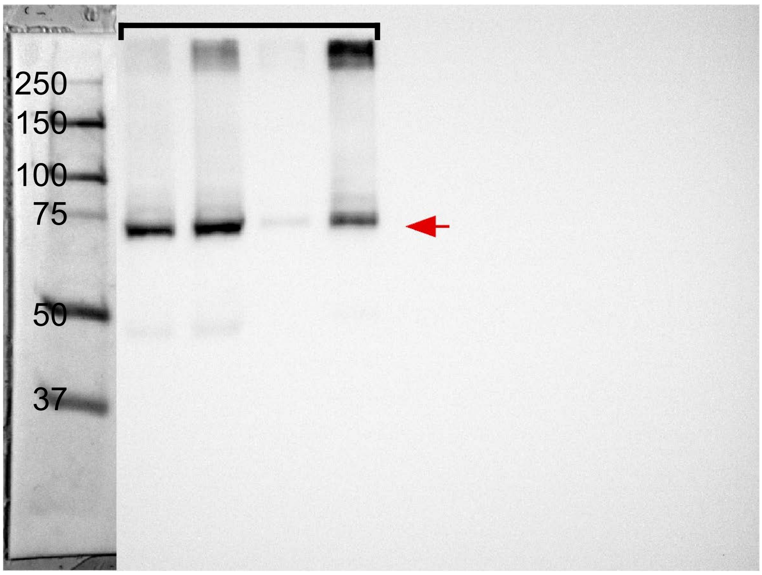

CD4ΔC-CALHM1 Cterm (+MG) (Na/K ATPase)

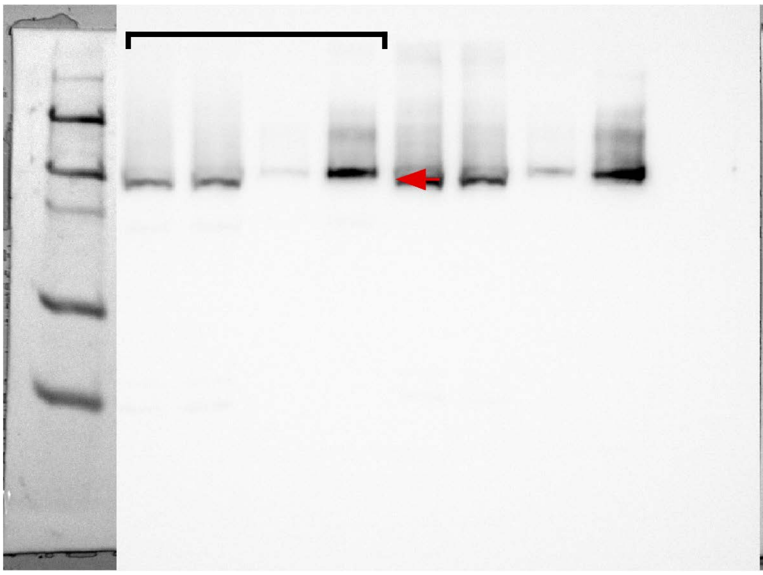

CD4ΔC-CALHM1 Cterm (+MG) (β-tubulin)

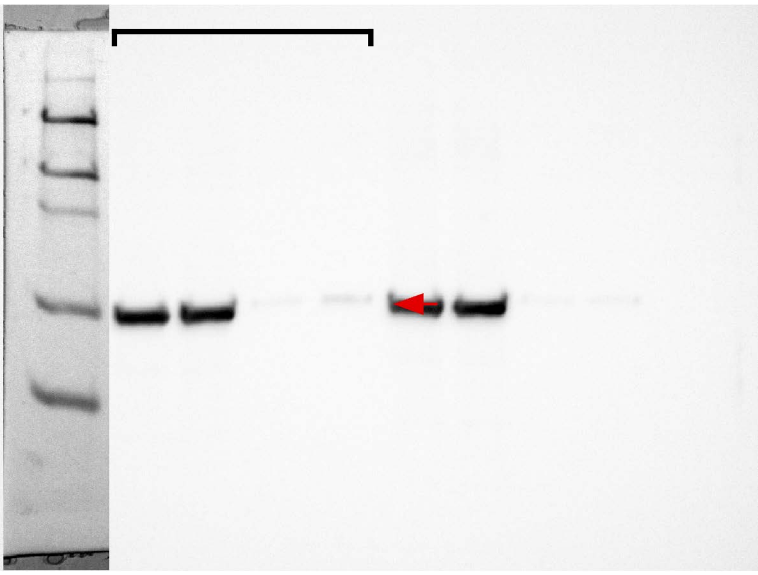

Supplementary figure 4
Uncropped blots for images shown in Fig. 7A

CD4ΔC-CALHM1 Cterm (YI/AA, +MG)
(FLAG)

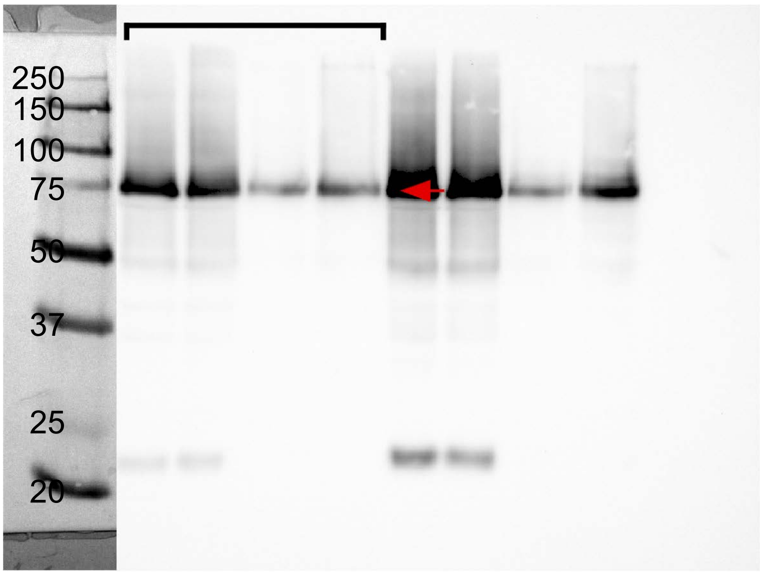

CD4ΔC-CALHM1 Cterm (YI/AA, +MG)
(Na/K ATPase)

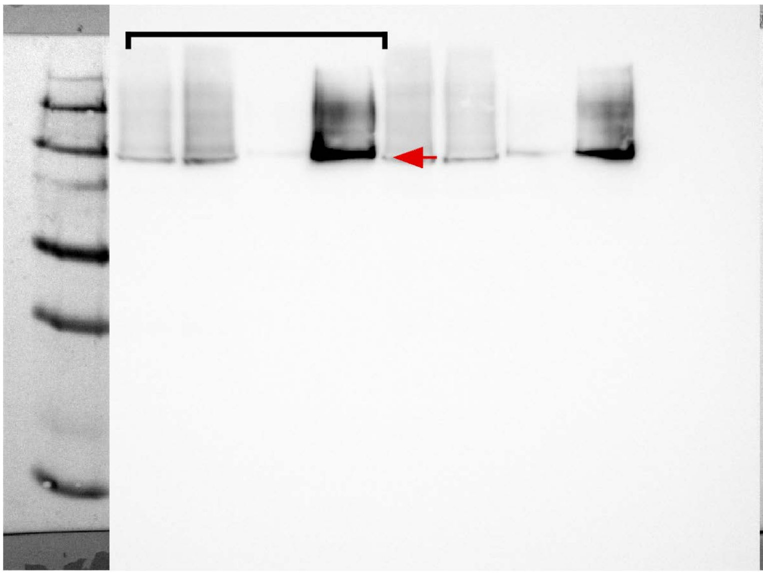

CD4ΔC-CALHM1 Cterm (YI/AA, +MG)
(β-tubulin)

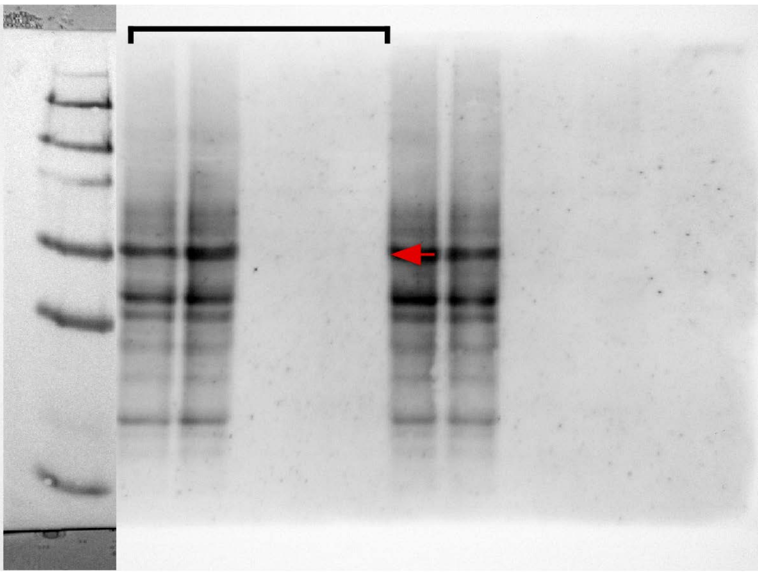

Supplementary figure 4
Uncropped blots for images shown in Fig. 7A

CD4ΔC-CALHM1 Cterm (LEL/AAA, +MG)
(FLAG)

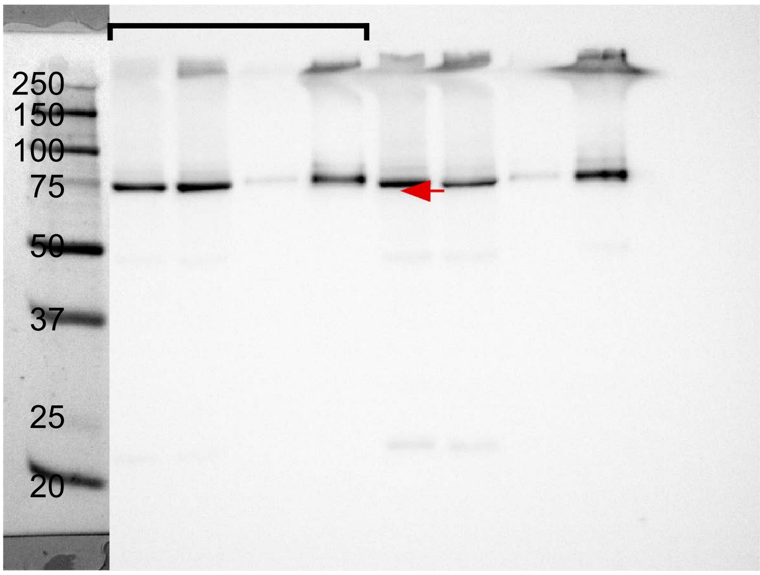

CD4ΔC-CALHM1 Cterm (LEL/AAA, +MG)
(Na/K ATPase)

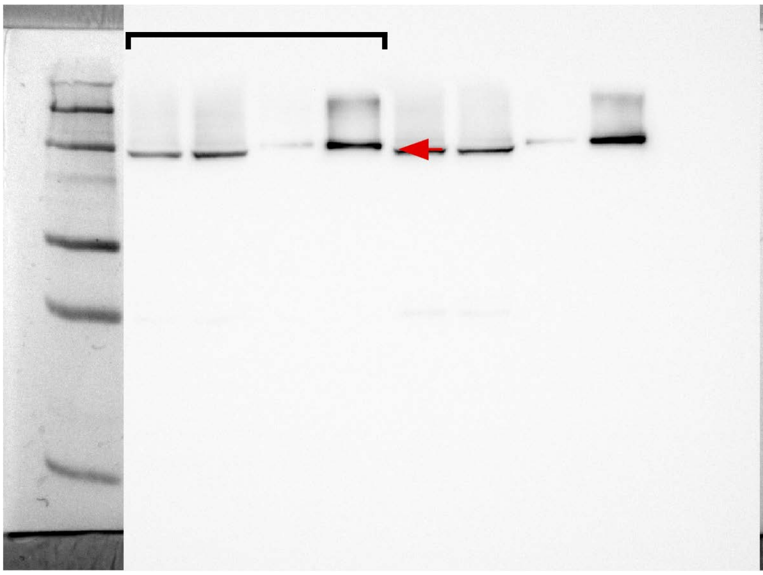

CD4ΔC-CALHM1 Cterm (LEL/AAA, +MG)
(β-tubulin)

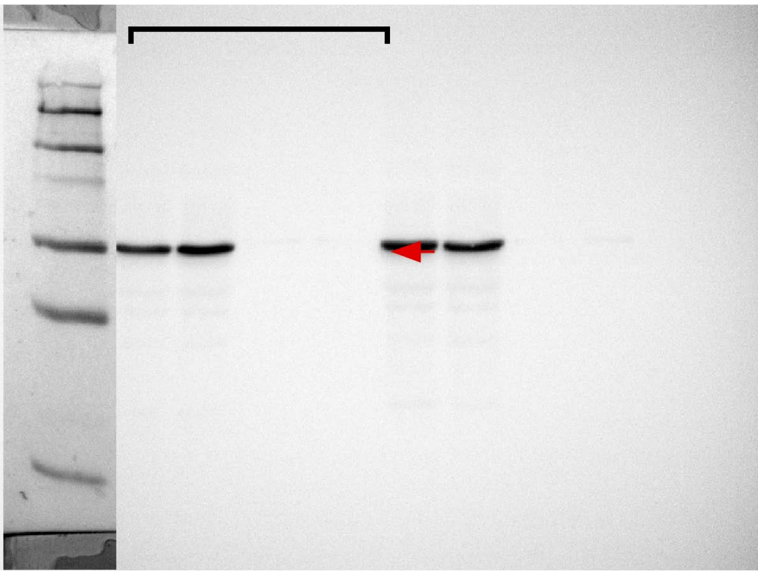

Supplementary figure 4
Uncropped blots for images shown in Fig. 7A

CD4ΔC-CALHM1 Cterm (LL/AA, +MG)
(FLAG)

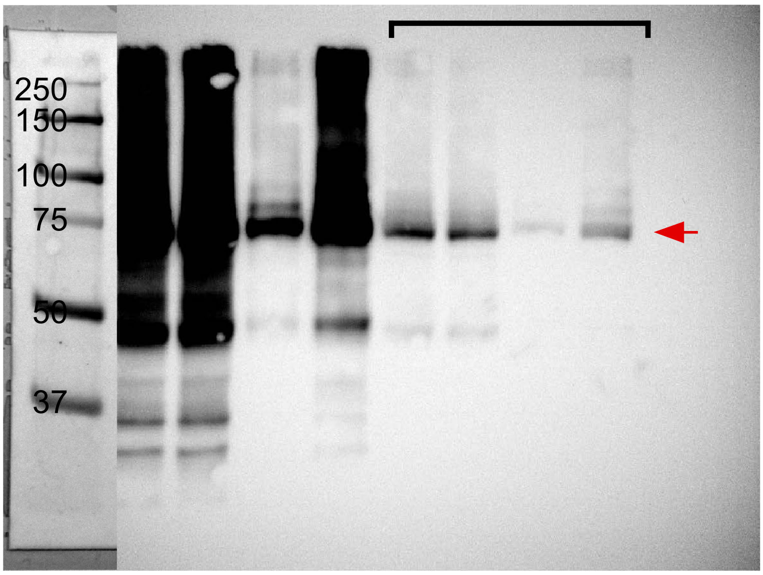

CD4ΔC-CALHM1 Cterm (LL/AA, +MG)
(Na/K ATPase)

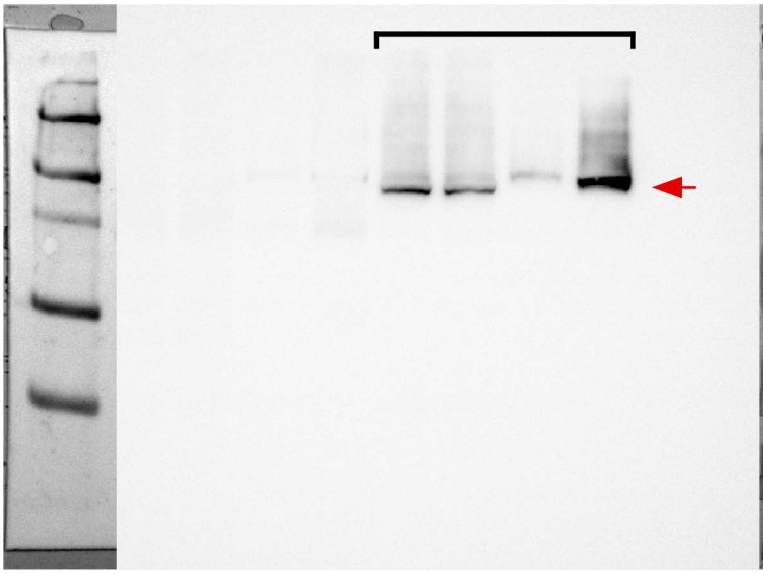

CD4ΔC-CALHM1 Cterm (LL/AA, +MG)
(β-tubulin)

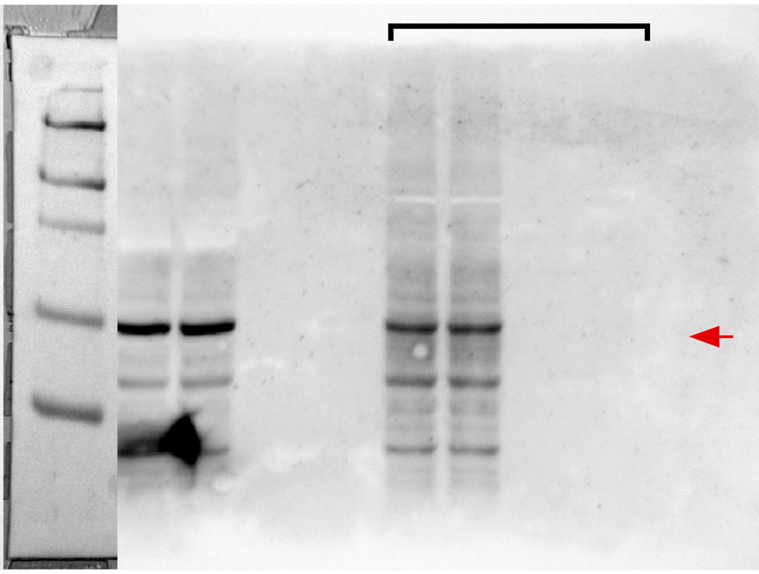

Supplementary figure 4
Uncropped blots for images shown in Fig. 7A

CD4ΔC-CALHM1 Cterm (LM/AA, +MG)
(FLAG)

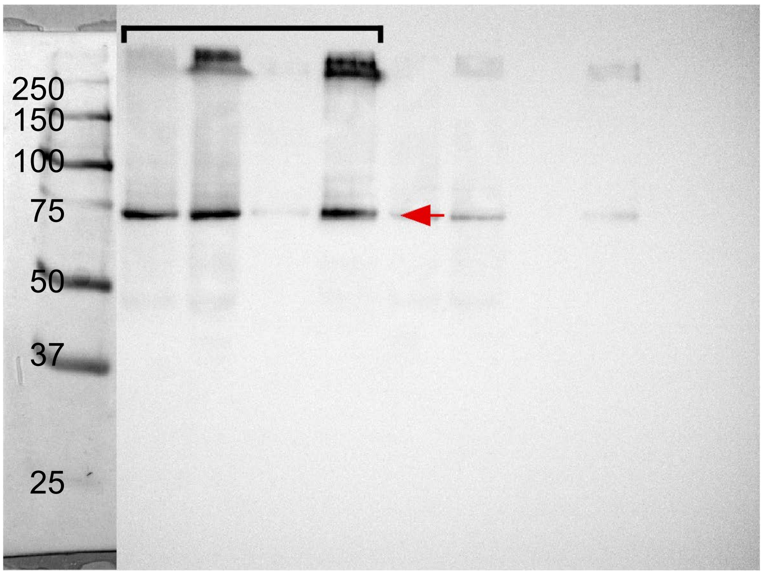

CD4ΔC-CALHM1 Cterm (LM/AA, +MG)
(Na/K ATPase)

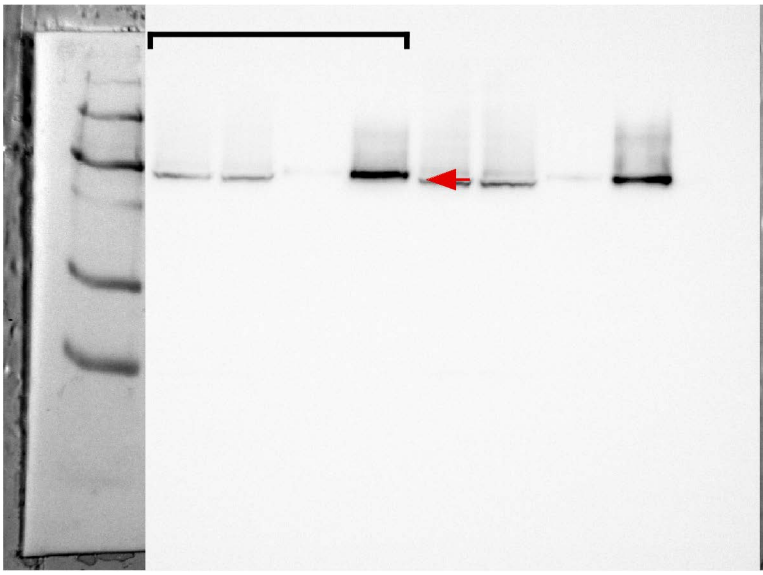

CD4ΔC-CALHM1 Cterm (LM/AA, +MG)
(β-tubulin)

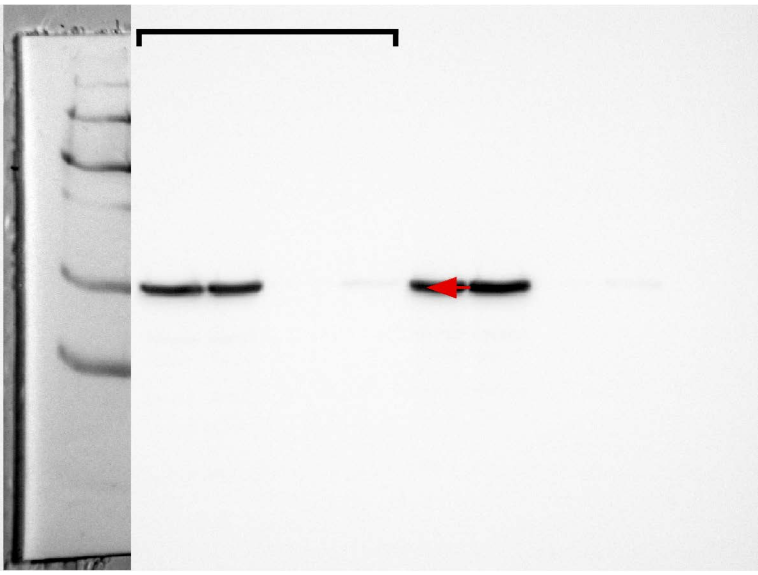

Supplementary figure 4
Uncropped blots for images shown in Fig. 7A

CD4ΔC-CALHM1 Cterm (PP/AA, +MG)
(FLAG)

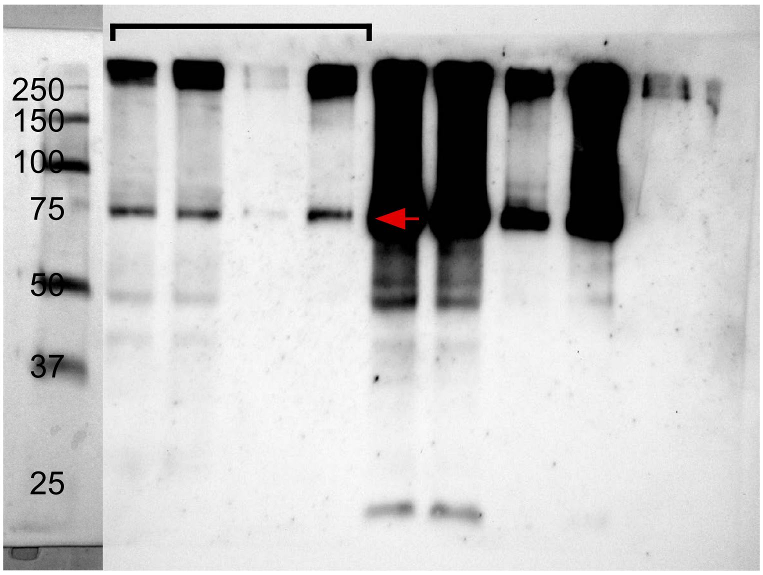

CD4ΔC-CALHM1 Cterm (PP/AA, +MG)
(Na/K ATPase)

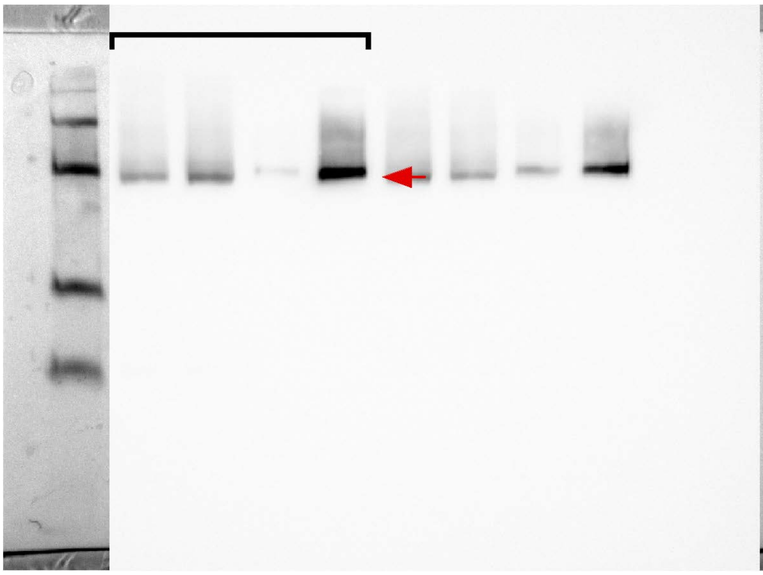

CD4ΔC-CALHM1 Cterm (PP/AA, +MG)
(β-tubulin)

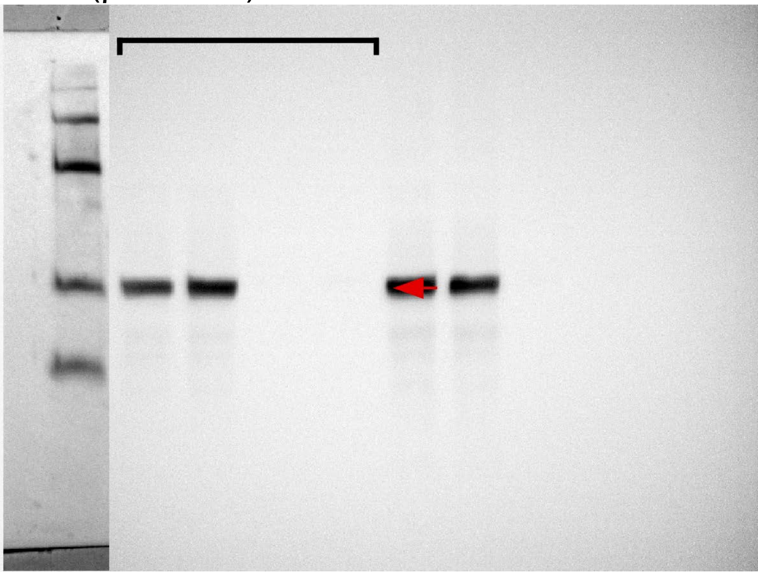

Supplement: Supplementary file 1 — Supplementary Information [file 41598_2019_39593_MOESM1_ESM.pdf]
